# An obesogenic feedforward loop involving PPARγ, acyl-CoA binding protein and GABA_A_ receptor

**DOI:** 10.1038/s41419-022-04834-5

**Published:** 2022-04-18

**Authors:** Gerasimos Anagnostopoulos, Omar Motiño, Sijing Li, Vincent Carbonnier, Hui Chen, Valentina Sica, Sylvère Durand, Mélanie Bourgin, Fanny Aprahamian, Nitharsshini Nirmalathasan, Romain Donne, Chantal Desdouets, Marcelo Simon Sola, Konstantina Kotta, Léa Montégut, Flavia Lambertucci, Didier Surdez, Grossetête Sandrine, Olivier Delattre, Maria Chiara Maiuri, José Manuel Bravo-San Pedro, Isabelle Martins, Guido Kroemer

**Affiliations:** 1 Centre de Recherche des Cordeliers, Equipe labellisée par la Ligue contre le cancer, Université de Paris, Sorbonne Université, Inserm U1138, Institut Universitaire de France, Paris, France; 2 Metabolomics and Cell Biology Platforms, Institut Gustave Roussy, Villejuif, France; 3 Faculté de Médecine, Université de Paris Saclay, Kremlin Bicêtre, France; 4 Department of Experimental and Health Sciences, Pompeu Fabra University (UPF), Barcelona, Spain; Center for Networked Biomedical Research in Neurodegenerative Diseases (CIBERNED), Madrid, Spain; 5 Laboratory of Proliferation, Stress and Liver Physiopathology, Centre de Recherche des Cordeliers, 75006 Paris, France; 6 Centre de Recherche des Cordeliers, INSERM, Sorbonne Université, Université de Paris, F-75006 Paris, France; 7 INSERM U1163, Institut Imagine, Paris, France; 8 INSERM U830, Équipe Labellisée LNCC, Diversity and Plasticity of Childhood Tumors Lab, PSL Research University, SIREDO Oncology Centre, Institut Curie Research Centre, 75005 Paris, France; 9 Bone Sarcoma Research Laboratory, Balgrist University Hospital, University of Zurich, Zurich, Switzerland; 10 INSERM U830, Équipe Labellisée LNCC, Diversity and Plasticity of Childhood Tumors Lab, PSL Research University, SIREDO Oncology Centre, Institut Curie Research Centre, 75005 Paris, France; 11 Unité de Génétique Somatique, Service d’oncogénétique, Institut Curie, Centre Hospitalier, 75005 Paris, France; 12 Facultad de Medicina, Departamento de Fisiología, Universidad Complutense de Madrid, Madrid, Spain; 13 Pôle de Biologie, Hôpital Européen Georges Pompidou, AP-HP, Paris, France

**Keywords:** Cell biology, Diseases

## Abstract

Acyl-coenzyme-A-binding protein (ACBP), also known as a diazepam-binding inhibitor (DBI), is a potent stimulator of appetite and lipogenesis. Bioinformatic analyses combined with systematic screens revealed that peroxisome proliferator-activated receptor gamma (PPARγ) is the transcription factor that best explains the ACBP/DBI upregulation in metabolically active organs including the liver and adipose tissue. The PPARγ agonist rosiglitazone-induced ACBP/DBI upregulation, as well as weight gain, that could be prevented by knockout of *Acbp*/*Dbi* in mice. Moreover, liver-specific knockdown of *Pparg* prevented the high-fat diet (HFD)-induced upregulation of circulating ACBP/DBI levels and reduced body weight gain. Conversely, knockout of *Acbp*/*Dbi* prevented the HFD-induced upregulation of PPARγ. Notably, a single amino acid substitution (F77I) in the γ2 subunit of gamma-aminobutyric acid A receptor (GABA_A_R), which abolishes ACBP/DBI binding to this receptor, prevented the HFD-induced weight gain, as well as the HFD-induced upregulation of ACBP/DBI, GABA_A_R γ2, and PPARγ. Based on these results, we postulate the existence of an obesogenic feedforward loop relying on ACBP/DBI, GABA_A_R, and PPARγ. Interruption of this vicious cycle, at any level, indistinguishably mitigates HFD-induced weight gain, hepatosteatosis, and hyperglycemia.

## Introduction

Obesity has become the worldwide most prevalent pathological condition and its comorbidities (including diabetes, cardiovascular disease, and cancer) account for an ever-increasing share of overall mortality [1, 2]. Although the etiology of obesity is complex, it appears that it constitutes a close-to-irreversible state, locking the increasingly unfit patient into a permanent habit of overeating. In spite of diets, exercise, lifestyle interventions, medications, and even surgical procedures, the vast majority of patients exhibit only transient weight loss followed by rebound effects. Thus, the National Weight Control Registry (which includes adults who have lost at least 13.6 kg of weight for a duration of at least 1 year, http://www.nwcr.ws) features only 10,000 members within a population of approximately 100 million obese in the United States (i.e., 1 among 10,000 obese individuals).

Multiple intertwined genetic, psychosocial, neuropsychiatric, neuroendocrine, metabolic, inflammatory, immune, and even microbiota-based circuitries have been invoked to contribute to obesity-associated food addiction [3–5]. Obviously, many studies have been designed to identify an increase in appetite-stimulatory factors—or a deficit in appetite-inhibitory factors—in obese subjects. Surprisingly, however, most established appetite-stimulatory factors (as exemplified by ghrelin) are actually reduced in obese persons [6], while most appetite-inhibitory factors (as exemplified by leptin) are increased in non-syndromic obesity [7], likely reflecting a state of failing homeostatic regulation [8]. Only a few appetite stimulators are genuinely elevated in obese individuals, as this is the case for acyl-coenzyme-A-binding protein (ACBP), which is encoded by the gene *diazepam-binding inhibitor* (*DBI*) [9–11].

ACBP/DBI (hereafter referred to as ACBP) is a phylogenetically ancient protein that is usually contained in the cytoplasm of nucleated cells, where it interacts with activated medium-chain fatty acids and participates in lipid metabolism [12, 13]. Being a leaderless peptide, it does not undergo conventional protein secretion However, it can be released into the extracellular space through an autophagy-associated pathway [9, 14]. Thus, mice kept without food for 1 to 2 days exhibit an autophagy-dependent increase in circulating ACBP protein levels [9], and human subjects experiencing voluntary fasting for several weeks (in a weight loss clinic) or involuntary fasting (due to cancer chemotherapy) show an elevation of plasma ACBP as well [11]. In starved mice, neutralization of ACBP by suitable antibodies strongly reduces refeeding, suggesting that ACBP acts as a bona fide appetite stimulator [9]. Indeed, intravenous injection of ACBP elicits feeding behavior, and its transgenic overexpression in the liver causes weight gain [10]. Similarly, in the nematode *Caenorhabditis elegans* and in the fruit fly *Drosophila melanogaster*, the orthologs of mammalian ACBP stimulate pharyngeal pumping and mouth hook movement, respectively, in line with the idea that ACBP is a phylogenetically ancient stimulator of food intake [15, 16]. Altogether, existing evidence suggests that, in mice, ACBP is embedded in a circuit, where starvation stimulates autophagy, thereby causing ACBP release from cells. Secreted ACBP acts on gamma-aminobutyric acid A receptors (GABA_A_R) expressed on multiple cell types outside of the central nervous system (e.g., cholangiocytes, hepatocytes, and macrophages) [17–19] to cause a transient decrease in glycemia and the consequent activation of orexigenic circuitries [9–11], thus stimulating food intake and closing a homeostatic feedback loop or “hunger reflex” [20].

Circulating ACBP levels are elevated in obese mice and humans. In humans, plasma ACBP correlates with body mass index (BMI), and elevated cardiometabolic risk factors, such as blood glucose, fasting insulin levels, total cholesterol, triglycerides, liver transaminases, and systolic blood pressure [9–11], as well as with signs of systemic inflammation [21]. Augmentation of plasma ACBP is associated with an obesity-associated upregulation of *ACBP* mRNA in the liver and white adipose tissue (WAT) (in mice and patients) and upregulation of ACBP protein in both liver and WAT from obese mice [9]. Conversely, human anorexia nervosa is accompanied by a reduction in ACBP plasma levels [9, 22]. Thus, it appears that long-term variations in BMI are coupled with a loss of the homeostatic “hunger reflex” giving rise to a permanent and pathogenic alteration of the setpoint of the system. In a hypothetical obesogenic feedforward loop, overeating would cause an increase in ACBP levels, which in turn favors excessive food intake [23].

Intrigued by the aforementioned hypothesis, we decided to identify the transcription factors (TFs) that best explain the obesity-associated alteration in gene expression profiles, including ACBP upregulation. Here, we report that peroxisome proliferator-activated receptor gamma (PPARγ) acts to transactivate the gene coding for ACBP in response to obesogenic stimuli, including high-fat diet (HFD) and PPARγ agonists. Intriguingly, ACBP also stimulates PPARγ activity through its action on GABA_A_R, hence completing a vicious amplification cycle that may contribute to the maintenance of the obese state.

## Materials and methods

### Chemicals, cell lines, culture conditions

Media and cell culture supplements were purchased from Gibco-Invitrogen (Carlsbad, CA, USA). Plasticware was purchased from Corning B.V. Life Sciences (Schiphol-Rijk, The Netherlands). Unless reported otherwise, all cell lines used in this study were cultured at standard conditions (37 °C, 5% CO_2_, Dulbecco’s modified Eagle’s medium containing 10% fetal bovine serum, 10 mM HEPES buffer, 100 U/mL penicillin G sodium, and 100 mg/mL streptomycin sulfate). Human hepatocellular carcinoma (HepG2, ATCC #HB-8065), murine hepatocellular carcinoma (Hep55.1c, ATCC), and murine hepatoma (Hepa1–6, ATCC #CRL-1830) cell lines were used for in vitro experimentation. Cell lines were tested negative for mycoplasma contamination. Cells were plated in 6-, 12-, or 9-well plates and grown for 24 h before treatment. HepG2 cells were treated with the PPARγ agonists rosiglitazone (2.5 μM, Sigma, St Louis, MO, USA, #R2408), edaglitazone (2.5 μM, Tocris Bio-Techne, Bristol, UK, #4784), GW1929 (2.5 μM, Sigma, St Louis, MO, USA, #G5668), S26948 (2.5 μM, Sigma, St Louis, MO, USA, #SML0510) daily and were harvested 8 days later to evaluate intracellular ACBP protein levels by cytofluorimetric assays. In addition, HepG2 cells were treated with the PPARγ agonists rosiglitazone (2.5 μM) and edaglitazone (5.0 μM), for 7 days followed by the *siRNA* transfection against *PPARG*. 96 h post-transfection, ACBP levels were quantified by cytometry.

### Stable *shAcbp*-expressing Hep55.1c cell line

shRNA targeting *Acbp* (*shAcbp-1*, TRCN0000317379; *shAcbp-2*, TRCN0000105052; *shAcbp-3*, TRCN0000105050), as well as negative control shRNA were cloned into the *pLKO.1-puro* lentiviral vector (Sigma, Burlington, MA, USA). About 2 × 10^5^ Hep55.1c cells were plated in a six-well plate (DMEM, 10% FBS) up to 60–70% confluence. Next, the cells were infected with lenti-shRNA particles (25–35 μL) in 1 mL fresh medium (DMEM, 10% FBS, 5 μg/mL Polybrene, Sigma, Burlington, MA, USA, # TR-1003). Twenty-four to forty-eight hours after transfection, the medium was replaced (DMEM, 10% FBS) and the cells were incubated for one additional day. The infected Hep55.1c cells were selected (10 µg/mL, puromycin, Thermo Fisher Scientific, Carlsbad, CA, USA, #A1113803) for 1 week. For monoclonal stable cell line isolation, single-cell sorting was performed according to standard protocols. The efficiency of lenti-shRNA against *Acbp* was determined by *Acbp* quantitative PCR and immunoblotting. For functional analysis, negative control and shRNA-*Acbp* Hep55.1c monoclonal lines were treated with rosiglitazone for 48 h followed by ACBP and PPARγ immunoblotting.

### Cytofluorometric assays

Cells were collected using Accutase (StemPro Accutase Cell Dissociation Reagent, Thermo Fisher Scientific, Carlsbad, CA, USA, #A1110501) and washed twice with PBS upon fixation with PBS at 2% PFA for 20 min at room temperature. Cells were permeabilized with 0.1% Triton X-100 for 10 min, washed twice with cold blocking solution (3% BSA, v/v in PBS), and stained overnight with primary antibodies at 4 °C. Cells were washed and incubated with secondary antibody AlexaFluor 647-conjugates in blocking buffer (60 min) and washed prior to flow cytometer analysis MACSQuant cytometer (Miltenyi Biotec, Bergisch Gladbach, Germany).

### Silencing RNA (*siRNA*) knockdown in vitro

*siRNAs* were transfected using Lipofectamine™ RNAiMAX Transfection Reagent (Invitrogen, Carlsbad, CA, USA, #13778150) according to the manufacturer’s instructions in presence of 100 nM of *siRNA* specific for *ACBP*, *ACOT1* (four sequences: *ACOT1*, *ACOT2*, *ACOT3*, *ACOT4*), *PPARG* (five sequences: *PPARG* Ambion, *PPARG* i, *PPARG* ii), and SMARTpool *siRNAs* against *CTCF*, *POLR2A*, *E2F1*, *SP1 NANOG*, *NFYA*, *EP300*, *FOS*, *NR3C1*, *CEBPB*, *MYC*, *FOXA1*, *FOXA2*, *TAF1*, *PHF8*, *JUND*, *MTA3*, *FOXP2*, *MBD4*, *PPARG,* and *SREBP* for 96 h incubation. The cells were treated as described and processed for downstream analyses. An empty vector or an unrelated *siRNA* (*UNR*) was used as a control.

### Immunoblotting

About 20–25 μg of protein lysates were separated by SDS-PAGE in 4–12% Bis-Tris acrylamide pre-cast gels (Thermo Fisher Scientific, Carlsbad, CA, USA, #WG1402BOX), and electro-transferred to immunoblot PVDF membranes (Biorad, Hercules, CA, USA, #1620177). To evaluate the electro-transfer efficiency, membranes were stained with Ponceau S solution, followed by rinsing and washing in TBST solution. Incubating the membranes in a blocking buffer for 2 h saturated unspecific binding sites. Blocking buffer was rinsed in TBST solution, and membranes were incubated overnight in the primary antibody (Human ACBP: Santa-Cruz #sc-376853, Mouse ACBP: Abcam #ab231910, Mouse FASN: Cell Signaling Technology #3180, Mouse GABRG2: Abcam # ab87328, Mouse GAPDH: Cell Signaling Technology #2118, Mouse PPARγ: Abcam #ab178860 & Santa-Cruz #sc-7273). Membranes were then washed in TBST followed by incubation in horseradish peroxidase (HRP)-labeled secondary antibody mix (Southern Biotech, Birmingham, USA). Membranes were finally washed in TBST solution and developed by the ImageQuant™ LAS 4000 (GE Healthcare) using SuperSignal West Pico chemiluminescent substrate (Thermo Fisher Scientific, Carlsbad, CA, USA, #34579).

### Co-immunoprecipitation assay

The physical interaction between the ACBP and the GABA_A_R γ2 subunit was examined by standard immunoprecipitation (IP) and immunoblotting protocols. In detail, liver protein extracts (500 μg) were immunoprecipitated on protein A/G-Sepharose beads (Merck Millipore, Burlington, MA, USA, #GE17–0618–01) using a goat polyclonal GABA_A_R γ2 antibody (3 μg per IP reaction, Abcam #ab240445) or its negative isotype control (IgG). Each IP reaction was incubated overnight (4 °C) in a rotation chamber followed by three consecutive rounds of PBS washing the next day. Each washing round included a PBS resuspension of the pellet and a re-centrifugation (12,000×*g*, 4 °C). Finally, beads were resuspended in 20 μL of NUPAGE 4x buffer (Life Technologies, CA, USA, #NP0008), heated at 100 °C (10 min), followed by standard immunoblotting for the GABA_A_R γ2 protein and the protein of interest.

### Mouse experiments

All mice used in this study were bred and housed in a pathogen-free, temperature-controlled environment with 12 h light/dark cycles according to the FELASA guidelines, EU Directive 63/2010, and French legislation. *Acbp*^*fl/fl*^, *Pparg*^*fl/fl*^, and *Gabrg2tm1Wul/J* 8–12-week-old male mice were bred in-house (CEF, Paris, France). *C57BL/6* 8–12-week-old male mice were purchased from Envigo (Envigo, Gannat, France). *B6.V-Lep*^*ob/ob*^*JRj* (obese), and *B6.V-Lep*^*ob/T*^*JRj* (lean) 8–12-week-old male mice were purchased from Charles River (Charles River Laboratory, Lentilly, France). All mice received a regular (Safe, #A04) or high-fat chow diet (Safe, #260 HF) and water ad libitum. For pharmacological studies, rosiglitazone, bexarotene (Sigma, St Louis, MO, USA, #SML0282), and HX531 (Sigma, St Louis, MO, USA, #SML2170) were administered intraperitoneally to *C57BL/6* mice at 6, 25, and 33 mg/kg body weight respectively on a daily basis over the course of 5 days. Unless reported otherwise, weight was monitored on a weekly basis. All animal experiments were conducted in compliance with the “C. Darwin” local Animal Experimental Ethics Committee (protocols #25355, # 10862, # 8530, #25010, # 0447.02, #25000).

### Adipocyte-specific *Acbp* knockout mice

Adipose tissue-specific *Acbp* knockout mice were generated as previously described [11]. In brief, *Acbp*^*fl/fl*^ mice (Bentley, WA, USA) were crossed with *B6;FVB-Tg*(*AdipoQ-cre*)*1Evdr/J* mice (Jackson Laboratory).

### Conditional whole-body *Pparg* knockout mice

Whole-body PPARγ knockout mice were generated by crossing *Pparg*^*fl/fl*^ mice (*Pparg*^*tm2Rev*^, loxP sites flanking exons 1 and 2; Bentley, WA, USA) with *B6.Cg-Tg* (*UBC-cre/ERT2*)*1Ejb/1* *J* mice (Jackson Laboratory, Bar Harbor, ME, USA). During breeding, genotype was verified by PCR using genomic DNA isolated from tail biopsies with primers specific for *Cre* and *Pparg* genetic loci.

*Pparg* primers:

1. *PpargF* (*oIMR1935*), 5′ TGGCTTCCAGTGCATAAGTT 3′
2. *PpargR* (*oIMR1934*), 5′ TGTAATGGAAGGGCAAAAGG 3′

PCR conditions: 95 °C (5 min); [95 °C (30 s), 60 °C (30 s), 72 °C (45 s)] x 35 cycles; 72 °C (5 min). PCR products were separated by electrophoresis in 2% agarose gels visualized using Ethidium bromide solution (Sigma, #E1510) or by using a capillary electrophoresis system LabChip^®^ GX Touch™ (PerkinElmer).

*Cre* primers:

1. *Cre1*, AGGTTCGTTCACTCATGGA;
2. *Cre2*, TCGACCAGTTTAGTTACCC.

PCR conditions: 95 °C (5 min); [95 °C (30 s), 60 °C (30 s), 72 °C (45 s)] x 35 cycles; 72 °C (5 min). PCR products were separated by electrophoresis in 2% agarose gels visualized using Ethidium bromide solution (Sigma, #E1510) or by using a capillary electrophoresis system LabChip^®^ GX Touch™ (PerkinElmer).

The expression of Cre recombinase was induced upon tamoxifen administration (75 mg/kg of body weight intraperitoneally on a daily basis over the course of 5 days). Tamoxifen was previously diluted in corn oil (90%) and ethanol (10%) up to the final concentration of 20 mg/ml followed by overnight incubation at 37 °C.

### GABA_A_R γ2 subunit mutated mice

*Gabrg2tm1Wul/J* 8–12-week-male mice were used in this study [24]. This genetic background encodes a point mutation (F77I) in the gamma-aminobutyric acid A Receptor (GABA_A_R) γ2 subunit (JAX™ Mice Strain, Charles River Laboratory, Lentilly, France) which, upon homozygosity, renders a compromised ACBP-GABA_A_R binding in a whole-body fashion.

During breeding, genotype was verified by PCR using genomic DNA isolated from tail biopsies with primers specific to the *Gabrg2* genetic locus.

*Gabrg2* primers:

1. *Gabrg2F (16697)*, 5′ AAGCGCCCACCTCTACTTCT 3′
2. *Gabrg2R (16698)*, 5′ TCATGGGATAGTGCATCAGC 3′

PCR conditions: 95 °C (5 min); [95 °C (30 s), 60 °C (30 s), 72 °C (45 s)] x 35 cycles; 72 °C (5 min). PCR products were separated by electrophoresis in 2% agarose gels visualized using Ethidium bromide solution (Sigma, #E1510) or by using a capillary electrophoresis system LabChip^®^ GX Touch™ (PerkinElmer).

### ACBP neutralization in vivo

Mice were injected intraperitoneally with mouse isotype IgG (negative control; 2.5 μg/g body weight, 200 μL) or monoclonal anti-ACBP-neutralizing antibody (2.5 μg/g body weight, 200 μL) twice per week over the course of 6 weeks (inVivoMAb #C1.18.4_BE0085 and Fred Hutch Antibody Technology #N/A respectively).

### Design of single-guide RNA (sgRNA) sequences and molecular cloning

sgRNA oligo-sequences (specific for *Pparg* exon 2: 5′ GTTCATGAGGCCTGTTGTAGAG 3′, and negative control *Rosa26* intron 1: 5′ GCTCGATGGAAAATACTCCGAG 3′) were designed (Broad Institute, portals.broadinstitute.org/gpp/public/analysis-tools/sgrna-design), synthesized (Eurogentec) and cloned in the *pX602-TBG* plasmid vector (BsaI restriction enzyme) (Addgene, #61593) [25]. Successful cloning was validated by DNA Sanger sequencing (Eurofins, U6 primer).

### Liver-specific CRISPR/Cas9 PPARγ knockout

*Rosa26* intron 1 genetic locus mutation served as negative a control (Loesch et al. bioRxiv, 2021). AAV8 vector (rAAV Platform, Imagine institute, France) was delivered to 4 weeks old male *C57/Bl6* mice via retro-orbital injection. All AAV8 doses were adjusted to 150 µL with a sterile physiological serum to a concentration of 2 × 10^11^ vg per mouse. Mice were randomized and attributed to the different experimental conditions. The mutation was validated in hepatocyte fraction at the DNA level (*Pparg* exon 2 or *Rosa26* loci PCR amplification, Sanger sequencing, Eurofins) and indel percentage was analyzed using the TIDE online software (https://tide.nki.nl/).

*Pparg* exon 2 primers:

1. *PpargF*, 5′ TTGTGCAGGTGAGGTTCTGG 3′
2. *PpargR*, 5′ ACAGACTCGGCACTCAATGG 3′

*Rosa26* intron 1 primers:

1. *Rosa26F*, 5′ CTTGCTCTCCCAAAGTCGCT 3′
2. *Rosa26R*, 5′ CCAATGCTCTGTCTAGGGGT 3′

PCR conditions: 95 °C (5 min); [95 °C (30 s), 55 °C (30 s), 70 °C (30 s)] x 30 cycles; 70 °C (10 min); 4 °C. PCR products were separated by electrophoresis in 2% agarose gels and visualized using SYBR Safe DNA gel stain (Invitrogen, Carlsbad, CA, USA, #S33102).

*Pparg* mutation was further validated at the protein level by immunoblot (Abcam, Cambridge, UK, #ab178860).

### Liver histology

Mouse liver samples were fixed in 20 mL 4% v/v formaldehyde solution (4 °C) for 24 h, followed by dehydration (incubation in gradually increasing ethanol solutions; 70–100% v/v) and paraffin inclusion. Five-micrometer sections were stained using hematoxylin and eosin (HE) and scanned by means of a Zeiss Lame Axioscan (objective: ×20). Images were analyzed using the Zen software.

### NAFLD score measurements

The NAFLD activity score was measured according to standard protocols. In brief, the sum of numerical scores describing steatosis (0–3), hepatocellular ballooning (0–2), and lobular inflammation (0–3) was calculated [26].

### ACBP and leptin detection in mouse plasma samples

Mouse blood was obtained from the submandibular vein using lithium heparin blood collection tubes (Sarstedt, France, #16443) followed by immediate centrifugation (12,000×*g*, 30 min, 4 ^o^C) for plasma isolation. Plasma was diluted 1:20 in ice-cold PBS and used as a template for ACBP (MyBioSource, #MBS2025156) and leptin (Merck Millipore, Burlington, MA, USA, #EZML-82K) quantification according to the manufacturer’s instructions.

### Food intake measurements

Eight-week-old male mice were individually housed and acclimatized in 25 × 15 × 10 cm cages followed by 6–8 h starvation (while receiving water ad libitum) prior to experimentation. The cumulative food intake was monitored over the course of 12 h.

### Blood glucose (glycemia) and β-hydroxybutyrate measurements

Fasted (12 h) blood glucose was measured using the Accu-Chek Performa machine (Accu-Check, France, #4702354). Fasted (12 h) blood β-hydroxybutyrate levels were measured using the Glucofix Ketone B reader (Menarini, Florence, Italy, #MEN45800).

### Metabolomic profiling (tissue and plasma) sample preparation

About 30 mg of the liver was harvested from each sacrificed mouse and placed in a 2-mL-homogenizer tube (Hard Tissue Homogenizing CK28, 2.8 mm zirconium oxide beads; Precellys, Bertin Technologies, France) containing 1 mL of ice-cold extraction mix (MeOH/water, 9/1, −20 °C, with a cocktail of internal standards). Samples were homogenized (three cycles of 20 s/ 5000 rpm; Precellys 24, Bertin Technologies, Montigny-le-Bretonneux, France) to facilitate solvent access and endogenous metabolites extraction, homogenates were centrifuged (10 min at 15,000×*g*, 4 °C), and finally supernatants were collected. Alternatively, plasma samples (25 μL) were mixed with 250 µL of the ice-cold extraction mixture, allowing protein precipitation and metabolites extraction, vortexed, and centrifuged (10 min at 15,000×*g*, 4 °C).

After sample centrifugation (either originating from organ tissue or plasma), supernatants were collected, separated into three fractions, and treated according to standard protocols [27]. Briefly, the first fraction was used for short-chain fatty acids downstream analysis (40 µL for both tissues and plasma samples), the second fraction was used for liquid chromatography/mass spectrometry (LC/MS) workflow and the third fraction was used for gas chromatography/mass spectrometry (GC/MS) workflow analyses. About 300 µL per tissue and 100 µL per plasma sample were transferred to an injection amber glass vial (with fused-in insert) and evaporated (Techne DB3, Staffordshire, UK) at 40 °C. The second dried fraction was recovered with 200 or 150 µL (tissue or plasma samples respectively) of ultra-pure water and stored at −80 °C until injection and analysis by LC/MS. The third dried fraction was derivatized before GC/MS injection and analysis. Finally, the fourth fraction and the sample pellet were re-extracted with an equal volume of 2% SSA (in MeOH), vortexed, and centrifuged (10 min at 15,000×*g*, 4 °C). The supernatant (350 and 60 µL, from tissue and plasma extracts respectively) was transferred to an injection polypropylene vial (with fused-in insert) and evaporated (Techne DB3, Staffordshire, UK) at 40 °C. Dried samples were recovered with ultra-pure water (200 and 100 µL, for tissue and plasma, dried extracts respectively) and stored at −80 °C until injection and analysis by (Ultra-high performance liquid chromatography/mass spectrometry) UHPLC/MS for polyamines detection.

### UHPLC/MS

Targeted UHPLC/MS analysis was performed using a UHPLC 1290 system (Agilent Technologies, Waldbronn, Germany) including an autosampler (4 °C), and a Peltier oven for rigorous control of the column temperature. The UHPLC was coupled to a triple quadrupole mass spectrometer (QQQ/MS) 6470 (Agilent Technologies) equipped with an electrospray source, using nitrogen as collision gas. Short-chain fatty acids and ketones bodies were detected in the first fraction by injecting 10 μL of sample into the Zorbax Eclipse XDB-C18 (100 mm × 2.1 mm, particle size 1.8 µm; Agilent) column protected by a guard column C18 (5 mm × 2.1 mm, particle size 1.8 μm). Column oven was maintained at 50 °C during analysis. The gradient mobile phase consisted of 0.01 % formic acid (Sigma) (A) and ACN (0.01% formic acid) (B). The flow rate was set to 0.7 mL/min, and the gradient was as follows: 20% B (initial conditions) maintained for 3 min, to 45% B in 4 min; then 95% B was maintained for 2 min, and finally equilibration to initial conditions, 20% B, for 1 min. The QQQ/MS was operated in negative mode. The gas temperature was set to 300 °C with a gas flow of 12 L/min. The capillary voltage was set to 5 kV.

For bile acid detection, 5 µL from samples recovered in water (second fraction) were injected into a Poroshell 120 EC-C8 (100 mm × 2.1 mm particle size 2.7 µm; Agilent technologies) column protected by a guard column (XDB-C18, 5 mm × 2.1 mm particle size 1.8 μm). The mobile phase consisted of freshly prepared 0.2% formic acid (A) and ACN/IPA (L/L; v/v) (B). The flow rate was set to 0.5 mL/min, and the gradient was as follows: 30% B increased to 38% B over 2 min; maintained for 2 min then increased 60% for 1.5 min, and finally to 98% B for 2 min (column washing), followed by 2 min of column equilibration at 30% B (initial conditions). The QQQ/MS was operated in negative mode. Gas temperature and flow were set to 310 °C and 12 L/min, respectively. The capillary voltage was set to 5 kV.

Polyamine profiling was performed using the fourth fraction: 10 μL of sample injection into a Kinetex C18 column (150 mm × 2.1 mm particle size 2.6 µm; Phenomenex) protected by a guard C18 column (5 mm × 2.1 mm, particle size 1.8 μm). The column oven was kept at 40 °C during analysis. The gradient mobile phase consisted of freshly prepared 0.1% HFBA (Sigma) (A) and ACN (0.1% HFBA) (B). The flow rate was set to 0.4 mL/min, as and gradient follows: from 5% (initial conditions) to 30% B in 7 min; then 90% B maintained 2 min, and finally equilibration to initial conditions (5% B, for 2 min). The QQQ/MS was operated in positive mode. The gas temperature was set to 350 °C with a gas flow of 12 L/min. The capillary voltage was set to 2.5 kV.

Tissue samples were injected for the analysis of nucleotides and co-factors into a Zorbax Eclipse plus C18 (100 mm × 2.1 mm, particle size 1.8 μm, Agilent) column protected by a guard column C18 (5 mm × 2.1 mm, particle size 1.8 μm). The column oven was kept at 40 °C during the analysis. The gradient mobile phase consisted of 0.5 mM DBAA (Sigma) (A) and ACN (B). The flow rate was set to 0.4 mL/min, and the gradient was as follows: 10% B (initial conditions) maintained for 3 min, increased to 95% B in 1 min, maintained for 2 min, to finally equilibrate to initial conditions, 10% B, for 1 min. The QQQ/MS was operated in both positive and negative modes. The gas temperature was set to 350 °C (gas flow: 12 L/min). The capillary voltage was set to 4.5 kV in positive mode and 5 kV in negative mode.

Multiple Reaction Monitoring (MRM) scan mode was used for targeted analysis in both GC and UHPLC/MS. Peak detection and integration were performed using the Agilent Mass Hunter quantitative software (B.10.1).

### Widely targeted analysis of intracellular metabolites

#### GC/MS

One microliter of derivatized samples (third fraction) was injected into a gas chromatograph (Agilent 7890B; Agilent Technologies, Waldbronn, Germany) coupled to a triple quadrupole mass spectrometer (QQQ/MS; 7000 C Agilent Technologies, Waldbronn, Germany), equipped with a high sensitivity electronic impact source (EI) operating in positive mode. The injection was performed in splitless mode. Front inlet temperature was kept at 250 °C, transfer line and ion-source temperatures were 250 and 230 °C, respectively. Septum purge flow was fixed at 3 mL/min. The purge flow to the split vent was operating at 80 mL/min for 1 min and gas saver mode was set to 15 mL/min after 5 min. Helium gas flowed through the column (HP-5MS, 30 m × 0.25 mm, i.d. 0.25 mm, d.f. J&WScientific, Agilent Technologies Inc.) at 1 mL/min. The column temperature was held at 60 °C for 1 min, raised to 210 °C (10 °C/min), then to 230 °C (5 °C/min), to finally reach 325 °C (15 °C/min), and held for 5 min. The collision gas was nitrogen.

### Pseudo-targeted analysis of intracellular metabolites

The metabolite profiling analysis was performed with a Dionex Ultimate 3000 UHPLC system (Thermo Fisher Scientific) coupled to an Orbitrap mass spectrometer (q-Exactive, Thermo Fisher Scientific) equipped with an electrospray source operating in both positive and negative mode, and acquired samples in full scan analysis mode (100–1200 m/z). LC separation was performed on the reversed-phase (Zorbax Sb-Aq 100 × 2.1 mm × 1.8 µm particle size), with mobile phases: 0.2 % acetic acid (A), and ACN (B). The column oven was maintained at 40 °C. Ten microliters of aqueous sample (second fraction) were injected for metabolite separation with a gradient starting from 2% B, increased to 95% B in 22 min, and maintained for 2 min for column rinsing, followed by column equilibration at 2% B for 4 min. The flow rate was set to 0.3 mL/min. The q-Exactive parameters were as follows: sheath gas flow rate 55 au, auxiliary gas flow rate 15 au, spray voltage 3.3 kV, capillary temperature 300 °C, S-Lens RF level 55 V. A sodium acetate solution was used to calibrate the mass spectrometer (dedicated to low mass calibration). Data were finally analyzed with the quantitative node of Thermo XcaliburTM (version 2.2) in a pseudo-targeted approach with a home-based metabolites list.

### Statistical analysis of metabolomic datasets

All targeted treated data were merged and cleaned with a dedicated R (version 3.4) package (@Github/Kroemerlab/GRMeta).

### RNA extraction, cDNA synthesis, gene expression analysis by quantitative PCR (qPCR)

Total RNA from HepG2 cells, liver tissues, and epididymal WAT was extracted using the RNeasy Plus Mini kit (Qiagen, Hilden, Germany, #74134), and RNeasy Lipid Tissue Mini kit (Qiagen, Hilden, Germany, #74804) respectively according to manufacturer’s instructions followed by reverse transcription (RT) for cDNA synthesis (Thermo Fisher Scientific, #11754050). Real-time-quantitative PCRs (RT qPCRs) were performed on a StepOnePlus Real-Time PCR System (Applied Biosystems) using TaqMan Gene Expression Master Mix (Applied Biosystems). Probes (mouse *Acbp*: #Mm01286585_g1, mouse *Pparg*: #Mm00440940_m1, mouse *Ppia*: #Mm02342430_g1, human *ACBP*: #Hs01554584_m1, human *PPARG*: #Hs01115513_m1, and human *GAPDH*: #Hs03929097_g1) were purchased from Thermo Fisher Scientific.

### RNA-sequencing library preparation

RNA was extracted from mouse livers using RNeasy Plus Mini Kit according to the manufacturer’s instructions followed by mRNA-sequencing library preparation (1.5 μg total RNA per sample; plant and animal eukaryotic strand-specific mRNA and sequencing). Sequencing was carried out on NovaSeq 6000 PE150 instrument (2 × 150 bp, 40 million reads per sample).

### RNA-sequencing data analysis

Pseudo-alignment and quantification were performed with the HISAT2 algorithm (reference genome *GRCm39*) [28]. Correlation analysis, principal component study, and differential expression analysis were performed with the DESeq2 package [29]. Differential gene expression analyses were done using the parametric Wald test with Benjamini-Hochberg adjustment (*p*_adj_). Genes with *p*_adj_ < 0.05 and a log2 fold change of ±1 were considered significantly differentially expressed genes. Gene set enrichment analysis (GSEA)-based gene ontology (GO) analysis, including biological process (BP) was performed on RNA-seq data from liver samples [30]. Graphs were constructed with a web-based bioinformatics tool (http://www.bioinformatics.com.cn).

### Chromatin extraction and PPARγ immunoprecipitation

Chromatin isolation from mouse livers was performed according to the manufacturer’s instructions (Diagenode, Liège, Belgium, #C01010055) followed by sonication and IP using anti-PPARγ (10 μg per IP, Abcam, #ab178860), and anti-H3K4me3 (1 μg per IP, Diagenode, #C01010055) antibodies. In brief, 0.15 g snap-frozen tissues were minced and cross-linked (1% of methanol-free formaldehyde; Thermo Fisher Scientific, #28908) for 6 min at room temperature followed by quenching (1/10 volume of 2 M glycine solution) for 2 min on ice. Isolated chromatin was sonicated (Diagenode, Bioruptor Pico) for 15 cycles (30 s “on”, 30 s “off”, position “high”) generating DNA fragments with an average size of ~150–300 bp.

### ChIP-sequencing library preparation

ChIP samples were eluted in 50 μL buffer C (Diagenode, #C01010055), 35 μL of which were used for the ChIP-sequencing (ChIP-seq) library preparation. Libraries were generated using the TruSeq ChIP library preparation kit (Illumina) and sequenced on Illumina NovaSeq 6000 (paired-end, 100 bp). Reads were aligned to a mouse reference genome (*mm10*) with bwa-mem 0.7.17-r1188 [31]. Uninformative reads (multi-mapped, duplicated, and low-mapping-score reads) were filtered out with samtools 1.3 [32]. Peaks were called with MACS2 2.1.1 [33] with the option narrow for PPARγ ChIP-seq and broad for H3K4me3 marks. For each sample, ChIP-seq was normalized according to their respective input DNA sample. The ChIP-seq signal tracks were generated using macs2 with bdgcmp (and –m FE to compute fold enrichment between the chromatin IP sample and the control). BedGraphToBigWig was utilized to convert the file to a binary format (BigWig).

### Chromatin immunoprecipitation quantitative PCR (ChIP qPCR)

ChIP samples were eluted in 50 μL according to the manufacturer’s instructions (Diagenode, #C01010055), then diluted 1:5 and, finally, used as a template for Real-Time qPCR (Power SYBR Green PCR Master Mix; Applied Biosystems, #4367659): DNA template 3 μL, H_2_O 2 μL, SYBR Green Master Mix 5 μL, [Oligonucleotides] = 10 μM 2 μL.

### Venn diagram

RNA-sequencing data were obtained from bovine adipocytes (GSE79347), murine hepatocytes (GSE77625), and human leukocytes (GSE145412) subjected to regular and high-fat regimens. Differentially expressed genes were assessed with Gene Expression Omnibus1 (refs. [34–36]), analyzed by the package DESeq25 [29], and subjected to upstream analyses based on the TRRUST software using a cut-off to exclude values between −1 and +1 [37].

### *ACBP* expression meta-analysis from GTEX and GEO datasets

Human (Genotype-Tissue Expression, GTEX), mouse (GEO), and rat (GEO) gene expression data were extracted. Bravais–Pearson correlation (R) of the expression between ACBP and various genes of interest was calculated on GEPIA2 (http://gepia2.cancer-pku.cn). Data were put in a heatmap using R packages ComplexHeatmaps and Circlize. Dendrogram consists of Complete-linkage clustering based on one euclidean distance matrix. The weighted *p* value is composed of mean R-value signs followed by the *p* value of the Student’s *t*-test with the null hypothesis (H0: µ = µ0 = 0).

### *ACBP* gene promoter binding motif analysis

ACBP promoter analysis using the Genome browser and ENCODE database allowed us to determine predictive TFs that could be implicated in *ACBP* expression regulation.

### Method details

All in vitro and in vivo experiments were replicated at least three times (*n* ≥ 3), which in our experience is optimal for obtaining significant results, with similar results. Data were reported as whisker plots (with each dot representing one biological replicate) including the mean ± SEM or as heatmaps. The sample size is noted in the figures. Normality tests and equal variance tests (F or Bartlett) were performed in the case of more than eight samples (*n* > 8). Statistical significance was analyzed using unpaired two-tailed Student’s *t*-test, unpaired two-tailed Student’s *t*-test with Welch’s correction, Mann–Whitney test, one-way ANOVA, or two-way ANOVA. Differences were considered statistically significant when *p* values were *p* < 0.05 or non-significant (ns) when *p* > 0.05. Immunoblot densitometric quantifications are reported as ratios of the protein band(s) of interest normalized over β-actin (unless otherwise indicated).

## Results

### PPARγ is the principal *ACBP* transactivator in obesity

**Fig. 1:**
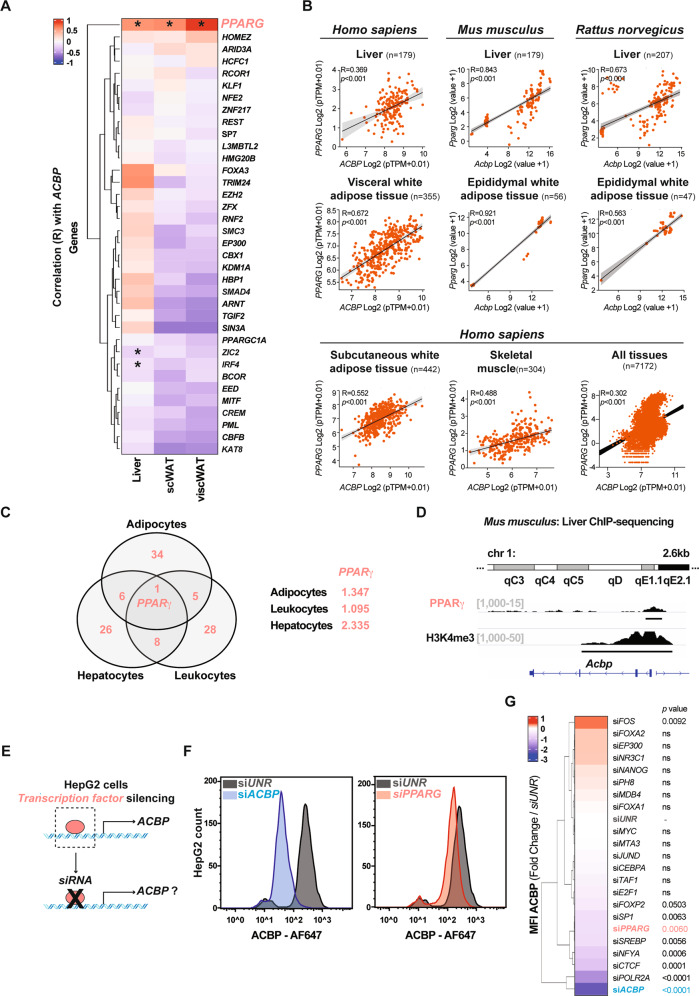
PPARγ transcription factor regulates the expression of *ACBP*. **A** Heatmap representation of correlation (R) between *ACBP* mRNA and mRNA of several genes in the human liver, subcutaneous white adipose tissue (scWAT), and visceral white adipose tissue (viscWAT) (*p < 0.05). **B** Correlation plots of *PPARG* and *ACBP* mRNA in liver and WAT from human (liver: *n* = 179, visceral WAT: *n* = 355), mouse (liver: *n* = 179, epididymal WAT: *n* = 56), and rat (liver: *n* = 207, epididymal WAT: *n* = 47) extracts. Correlation plots of *PPARG* and *ACBP* mRNA in subcutaneous white adipose tissue (*n* = 442), skeletal muscle (*n* = 304), and the aggregate of all tissues (*n* = 7172) from human origin. **C** Venn diagram representation includes transcription factors (TFs) the targets of which are upregulated in bovine adipocytes, murine hepatocytes, and human leukocytes when their donors receive a high-fat diet (left). Significance of the upregulation of PPARγ target genes in each of the three datasets (right). **D** Chromatin immunoprecipitation sequencing (ChIP-seq) signals of PPARγ and H3K4me3 (*Acbp* promoter, mouse liver, *n* = 3). The black line corresponds to the peaks called per MACS. **E** Silencing of TFs encoding human *ACBP* in HepG2 cells. **F** Cytofluorometric peaks quantifying ACBP after silencing the unrelated negative control, *ACBP*, or *PPARG* (*siUNR*, *siACBP*, or *siPPARG*). **G** Heatmap representation of cytofluorometric ACBP protein levels upon silencing various TFs encoding *ACBP* in HepG2 cells (*n* = 3; one-way ANOVA). For statistical analyses (**A**, **B**) *p* values and R were calculated by Pearson and Spearman correlations respectively. See also Fig. S1.

A bioinformatic analysis designed to uncover which TFs best correlate with the *ACBP* mRNA expression in major metabolic tissues (human liver, subcutaneous and visceral WAT) indicated that *PPARG* mRNA levels best correlate with those of *ACBP* (GEPIA2, GTEX databases, Fig. 1A, B). *ACBP* also correlated with *PPARG* in numerous other tissues of human origin (Fig. 1B and Fig. S1A), as well in tissues from rodents including mice (Fig. 1B and Fig. S1B) and rats (Fig. 1B and Fig. S1C), suggesting interspecies conservation. This applies to the liver and white adipose tissue from humans and rodents, as well as to human subcutaneous adipose tissue, skeletal muscle, and the aggregate of all tissues (Fig. 1B). RNA-sequencing (RNA-seq) data from bovine adipocytes, murine hepatocytes, and human leukocytes were analyzed to identify TFs the target genes of which are modulated under a normal or obesogenic diet. Only the target genes of one single TF, PPARγ, were activated consistently across these three cell types and species under obesogenic conditions (Fig. 1C). For this reason, we decided to focus on PPARγ. Chromatin immunoprecipitation (ChIP) sequencing of PPARγ-associated chromatin in mouse liver confirmed that PPARγ binds to the promoter of *Acbp* within a region bearing the euchromatin marker H3K4me3 (Fig. 1D). Moreover, knockdown of multiple TFs (and DNA-binding proteins) predicted to bind to the *ACBP* promoter (Fig. S1D) in human HepG2 cells of hepatocellular origin confirmed that PPARγ is required for the baseline expression of *ACBP* mRNA and protein (Fig. 1E–G and Fig. S1E).

Altogether, these results confirm prior reports indicating that PPARγ can transactivate ACBP [38, 39] and support the notion that PPARγ is an obesogenic TF that stimulates *ACBP* expression.

### ACBP and PPARγ induce each other

**Fig. 2:**
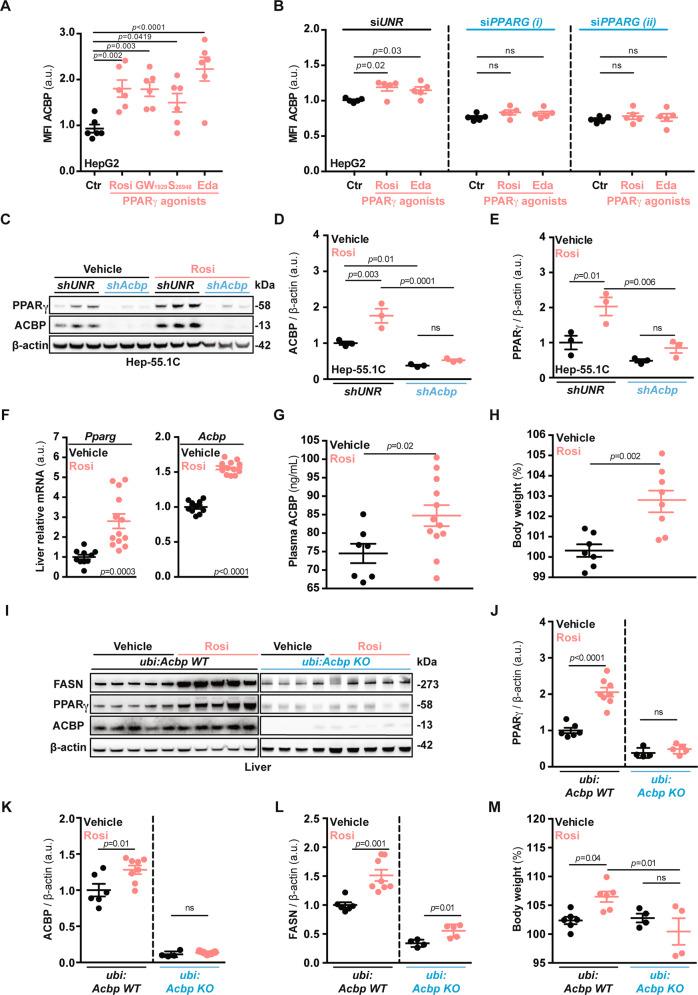
The effects of PPARγ-modulating agents depend on ACBP function. **A** Cytofluorometric measurement of ACBP protein after treatment with PPARγ agonists (Ctr: vehicle, Rosi: rosiglitazone, GW1929, S26948, Eda: edaglitazone) (*n* = 6) in control (*siUNR*) or *PPARG*-silenced (*siPPARG*) HepG2 cells (*n* = 5) (**B**) (MFI: mean fluorescence intensity normalized to control). **C** Representative immunoblot images of PPARγ, ACBP, and β-actin proteins in control (*shUNR*) and *Acbp*-knocked down (*shAcbp*) Hep55.1c cells after treatment with vehicle or Rosi (48 h), densitometric quantification (*n* = 3) (**D**, **E**). **F** *Pparg* and *Acbp* mRNA expression measurements in liver extracts obtained from mice receiving Rosi or vehicle (5 days) (*n* = 10 to 13 mice per condition). **G** Plasma ACBP concentration (*n* = 7 to 12 mice per condition), and **H** body weight measurements from mice receiving Rosi or vehicle (5 days) (*n* = 7 to 8 mice per condition). **I** Liver representative immunoblot images of FASN, PPARγ, ACBP, and β-actin proteins from ACBP-control (*ubi:Acbp WT*) or ACBP knockout (*ubi:Acbp KO*) mice receiving vehicle or Rosi (5 days), densitometric quantification (*n* = 4 to 8 mice per condition) (**J**–**L**). **M** Body weight measurements from mice administrated with Rosi or vehicle (2 months) (*n* = 4 to 6 mice per condition). Results are displayed as whisker plots (with each dot representing one in vitro biological replicate or one single mouse) including the mean ± SEM. For statistical analyses, *p* values (indicating statistical comparisons with the control condition) were calculated by a two-tailed unpaired Student’s *t*-test. For statistical analysis *p* values were calculated by two-tailed unpaired Student’s *t*-test (**G**, **H**, **J**–**L**) applying Welch correction (**F**), one-way ANOVA (**A**, **B**), or two-way ANOVA (**D**, **E**, **M**). MFI mean fluorescence intensity, a.u. arbitrary units, kDa kilodaltons, sh short-hairpin, ubi ubiquitous, ns non-significant. See also Fig. S2.

**Fig. 3:**
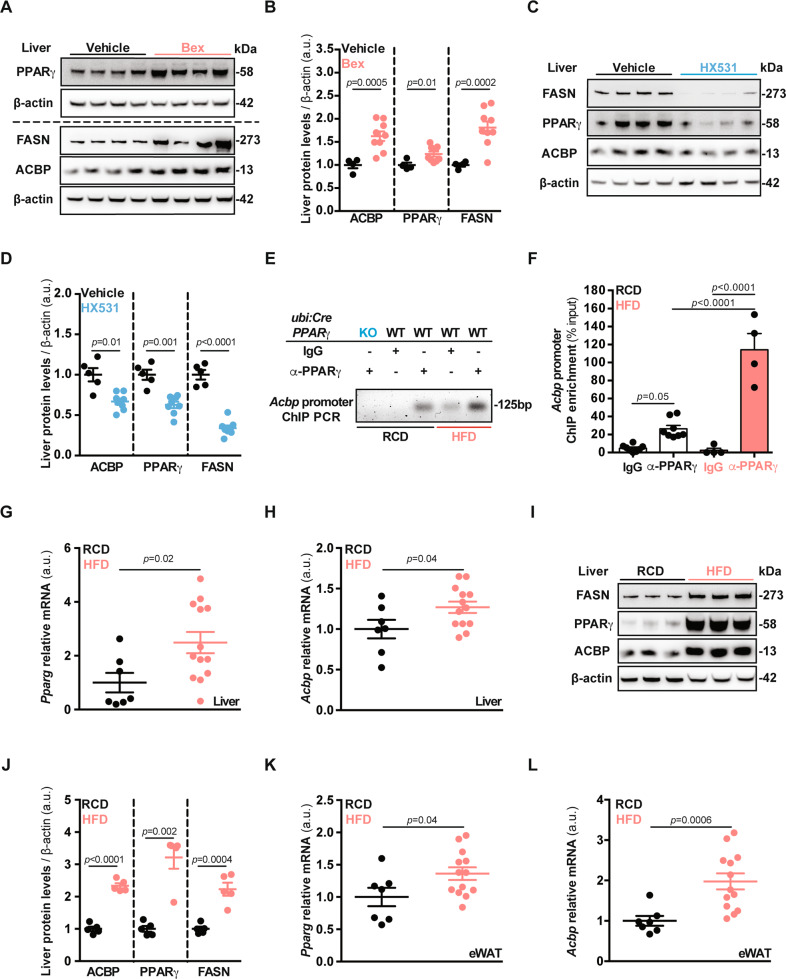
Pharmacological and dietary PPARγ manipulations regulate the expression of *Acbp*. **A** Liver representative immunoblot images of PPARγ, ACBP, FASN, and β-actin proteins from mice receiving control (Vehicle), or RXRα agonist Bexarotene (Bex), densitometric quantification (*n* = 4 to 9 mice per condition) (**B**). **C** Liver representative immunoblot images of PPARγ, ACBP, FASN, and β-actin proteins from mice receiving control (Vehicle), or RXRα antagonist HX531 drugs (5 days), densitometric quantification (*n* = 4 to 8 mice per condition) (**D**). **E** Qualitative α-PPARγ chromatin immunoprecipitation (ChIP) analysis from liver extracts obtained from mice receiving regular-chow (RCD) or high-fat diet (HFD). ChIP PCR products in the case of DNA templates originated from chromatin samples that have been precipitated with a PPARγ-specific antibody (α-PPARγ). No product in chromatin samples precipitated with negative isotype control (IgG). No ChIP PCR product in the α-PPARγ sample originating from the whole-body PPARγ knockout mice (*ubi:Cre* PPARγ KO) (*n* = 3 per condition). **F** Quantitative analysis of ChIP Real-Time PCR (*n* = 4 to 8 mice per condition). **G**, **H** Liver and **K**, **L** epididymal white adipose tissue (eWAT) *Pparg* and *Acbp* mRNA expression measurements obtained from mice receiving RCD or HFD (6 weeks) (*n* = 7 to 13 mice per condition). **I** Liver representative immunoblot images of FASN, PPARγ, ACBP, and β-actin proteins from mice receiving RCD or HFD (6 weeks), densitometric quantification (*n* = 5 mice per condition) (**J**). Results are displayed as whisker plots (with each dot representing one single mouse) including the mean ± SEM. For statistical analysis *p* values were calculated by two-tailed unpaired Student’s *t*-test (**G**, **H**, **K**, **L**) applying Welch correction (**B**, **D**, **J**), or two-way ANOVA (**F**). kDa kilodaltons, a.u. arbitrary units, bp base pairs, ns non-significant. See also Fig. S3.

Several pharmacological PPARγ agonists including antidiabetic thiazolidinediones (rosiglitazone, edaglitazone) as well as chemically unrelated tool compounds (GW1929, S26948) elevated ACBP protein levels in human HepG2 cells (Fig. 2A). In line with these results, culture of mouse Hepa1–6 and Hep55.1c hepatoma cells with rosiglitazone led to increased ACBP levels (Fig. S2A–C). The thiazolidinedione-induced ACBP upregulation was reversed by knocking down *PPARG* (Fig. 2B), demonstrating the specificity of this treatment. Similar results were obtained in Hep55.1 C cells, in which rosiglitazone induced an increase in both PPARγ and ACBP proteins, and this effect was reversed by knockdown of *Acbp* (Fig. 2C–E). Short-term (5 days) treatment of mice with daily intraperitoneal (i.p.) injections of rosiglitazone caused an increase in *Pparg* and *Acbp* expression in the liver (Fig. 2F) and epididymal WAT (Fig. S2D–F), as well as an elevation in plasma ACBP concentrations (Fig. 2G), coupled to a minor (by 3%) but significant (*p* = 0.002, unpaired Student *t*-test) increase in body weight (Fig. 2H). Similarly, after long-term (2 months) treatment with rosiglitazone, mice exhibited an increase in protein expression of PPARγ, ACBP, and fatty acid synthase (FASN), which is another PPARγ target gene [40, 41] (Fig. 2I–L). Of note, all these rosiglitazone-induced changes, including the weight gain, were abolished upon inducible whole-body ACBP knockout (Fig. 2I–M). Short-term (5 days) treatment with bexarotene (an agonist of retinoid X receptor α, RXRα, a coactivator of PPARγ, Fig. S3A) also induced PPARγ, ACBP, and FASN proteins in the liver (Fig. 3A, B) while the RXRα inhibitor HX531 showed the opposite effects (Fig. 3C, D). These in vivo pharmacological assays underscore the co-regulation between PPARγ and ACBP.

In agreement with our recent findings [11], plasma ACBP levels were positively correlated with body weight gain during a short course (1 month) of HFD (Fig. S3B, C). This lipoanabolic regimen favored the binding of PPARγ to the *Acbp* promoter in vivo, as demonstrated by PPARγ chromatin immunoprecipitation (ChIP) followed by quantitative reverse transcriptase PCR (ChIP-qRT PCR) (Fig. 3E, F and Fig. S3D, E). This HFD effect was accompanied by an increase in total *Pparg* and *Acbp* mRNA (Fig. 3G, H) and protein levels in the liver (Fig. 3I, J) as well as in epididymal white adipose tissue extracts (eWAT, Fig. 3K, L).

Collectively, these results indicate that ACBP and PPARγ are co-regulated in several tissues, in response to PPARγ agonists, RXR modulators as well as HFD.

### PPARγ removal suppresses HFD-induced ACBP induction and vice versa

**Fig. 4:**
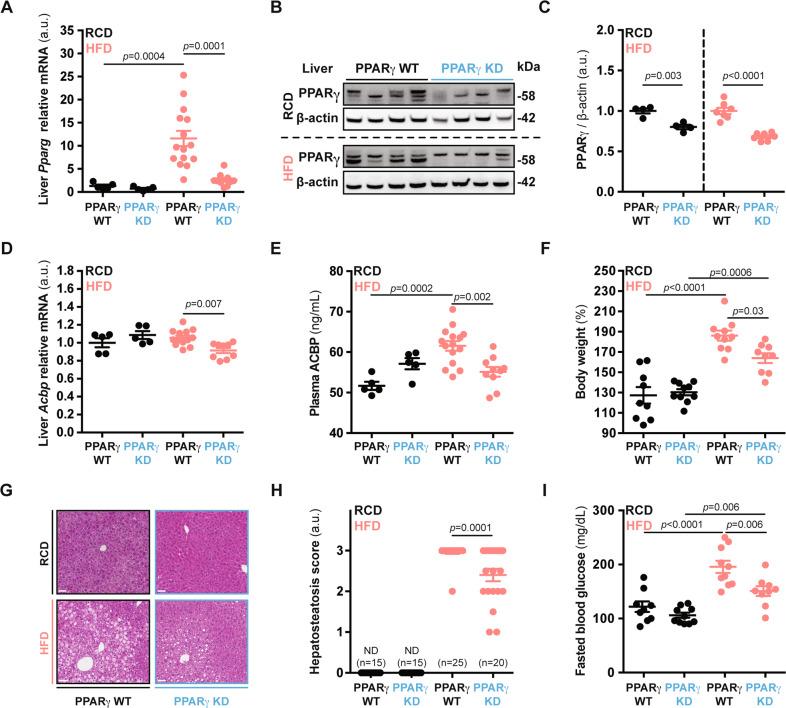
Liver-specific PPARγ knockout yields decreased ACBP levels. **A** *Pparg* mRNA expression (*n* = 5 to 15 mice per condition) and **B**, **C** protein level measurements (*n* = 4 to 7 mice per condition) in liver extracts obtained from control (PPARγ WT) or liver-specific CRISPR/Cas9-mediated PPARγ-knockdown mice (PPARγ KD) receiving regular-chow (RCD) or high-fat diet (HFD). For statistical analysis (**C**) *p* values were calculated comparing HFD groups to the corresponding RCD group for each genetic background (PPARγ WT, PPARγ KD). **D** *Acbp* mRNA expression (*n* = 5 to 13 mice per condition), **E** plasma ACBP concentration (*n* = 5 to 15 mice per condition), and **F** body weight gain measurements in control or PPARγ KD mice rendered obese (HFD, 2 months) (*n* = 9 to 10 mice per condition). **G** Representative hematoxylin eosin (HE) images of control or PPARγ KD livers (*n* = 15 to 25 mice per condition) obtained from mice receiving RCD or HFD, hepatosteatosis score quantification (bar: 50 μm, ND non-detected) (**H**). **I** Fasted blood glucose concentration from control or liver-PPARγ KD mice receiving RCD or HFD (*n* = 9 to 10 mice per condition). Results are displayed as whisker plots (with each dot representing one single mouse) including the mean ± SEM. For statistical analysis *p* values were calculated by two-tailed unpaired Student’s *t*-test (**C**), two-way ANOVA (**A**, **D**–**F**, **I**), or Mann–Whitney test (**H**). kDa kilodaltons, a.u. arbitrary units, ns non-significant, ND non-detected. See also Fig. S4.

In the next step, we suppressed *Pparg* expression in the liver by employing a hepatotropic adeno-associated viral vector (AAV8) carrying a plasmid encoding the Cas9 protein and *Pparg*-specific single-guide RNAs (sgRNAs) (Fig. S4A). The resulting hepatocyte-specific *Pparg* gene knockout led to an overall decrease of *Pparg* mRNA and protein, both in livers from mice receiving a regular-chow diet (RCD) and in livers from mice subjected to an HFD regimen—that would have otherwise manifested an upregulation of PPARγ (Fig. 4A–C). PPARγ deletion reduced the HFD-induced increase in *Acbp* liver mRNA and ACBP plasma protein (Fig. 4D, E). This was coupled with a reduction of HFD-induced body weight gain (Fig. 4F), attenuated hepatic steatosis (Fig. 4G, H), decreased local accumulation of HFD-induced fatty acids (Fig. S4B–E), and reversal of HFD-induced hyperglycemia (Fig. 4I).

**Fig. 5:**
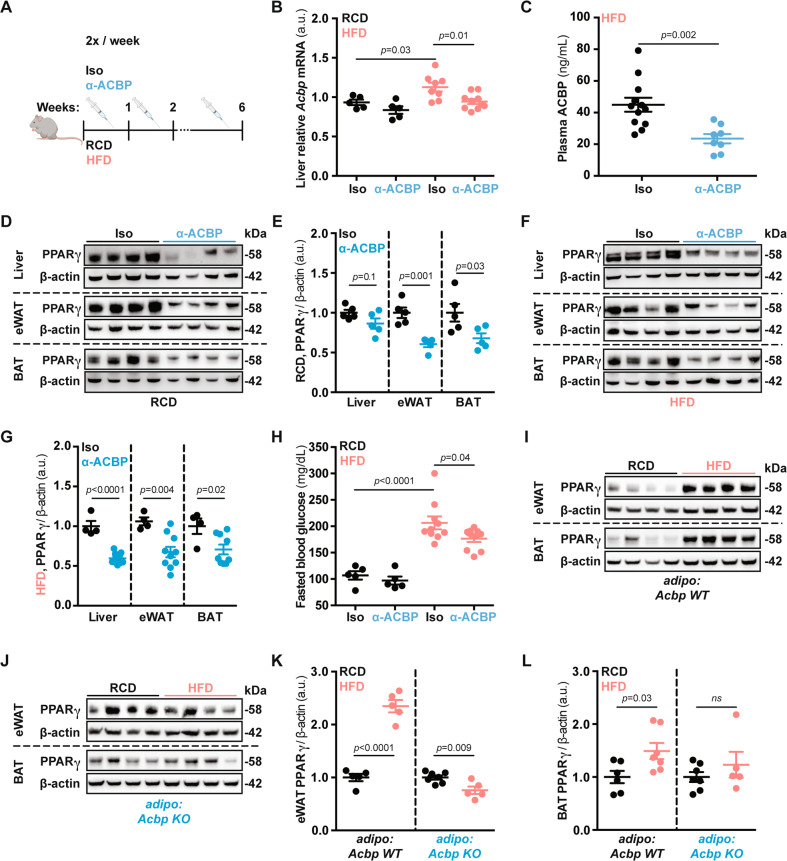
Neutralization or genetic ablation of ACBP results in decreased PPARγ. **A** ACBP-neutralizing antibody (α-ACBP) or isotype control (Iso) was administered in vivo by intraperitoneal injection in mice fed with regular-chow (RCD) or high-fat diet (HFD). **B** *Acbp* mRNA expression measurement in liver extracts obtained from mice receiving Iso or α-ACBP in RCD or HFD feeding regimens (*n* = 5 to 9 mice per condition). **C** Plasma ACBP concentration measurement in Iso—or α-ACBP – treated mice (*n* = 8 to 10 mice per condition). **D**, **F** Liver, epididymal white adipose tissue (eWAT), and brown adipose tissue (BAT) representative immunoblot images of PPARγ and β-actin proteins from mice receiving Iso or α-ACBP (RCD or HFD), densitometric quantification (RCD: *n* = 5 to 10 mice per condition, HFD: *n* = 4 to 10 mice per condition) (**E**, **G**). **H** Fasted blood glucose concentration from mice receiving Iso or α-ACBP (RCD or HFD) (*n* = 5 to 10 mice per condition). **I**, **J** eWAT and BAT representative immunoblot images of PPARγ and β-actin proteins from adipocyte-specific ACBP knockout murine model (*adipo: Acbp KO*) compared to control (*adipo: Acbp WT*), densitometric quantification (*n* = 5 to 7 mice per condition) (**K**, **L**). Results are displayed as whisker plots (with each dot representing one single mouse) including the mean ± SEM. For statistical analysis *p* values were calculated by two-tailed unpaired Student’s *t*-test (**C**, **E**, **G**, **K**, **L**) or two-way ANOVA (**B**, **H**). a.u. arbitrary units, ns non-significant, kDa kilodaltons. See also Fig. S5.

Next, we injected a neutralizing anti-ACBP antibody (Fig. 5A) that reduced the level of *Acbp* liver mRNA expression, as well as circulating ACBP protein in the context of HFD (Fig. 5B, C). ACBP neutralization also reduced PPARγ protein expression in the liver, eWAT, and brown adipose tissue (BAT), both in mice subjected to an RCD (Fig. 5D, E) and in mice subjected to HFD (Fig. 5F, G). In agreement with our previous findings [9], systemic ACBP neutralization reduced signs of non-alcoholic fatty liver disease (NAFLD) including local inflammation (Fig. S5A–C), and ameliorated the HFD-induced hyperglycemic effect (Fig. 5H), while increasing the ketone body 3-hydroxybutyrate (Fig. S5D). Of note, ACBP neutralization decreased the HFD-induced plasma leptin levels (Fig. S5E). We also determined the effects of the adipocyte-specific knockout of ACBP (Fig. S5F–H) that confers resistance to HFD-induced obesity [10]. Removal of ACBP in adipocytes abolished the HFD-induced upregulation of PPARγ, both in epididymal WAT and in BAT (Fig. 5I–L).

In conclusion, genetic removal of PPARγ abolishes HFD-induced ACBP upregulation, while vice versa neutralization or knockout of ACBP prevented HFD-induced PPARγ upregulation. Altogether, these results confirm the existence of an HFD-triggered positive feedback loop between ACBP and PPARγ.

### Mutation of the ACBP binding domain in GABA_A_R confers resistance to HFD

**Fig. 6:**
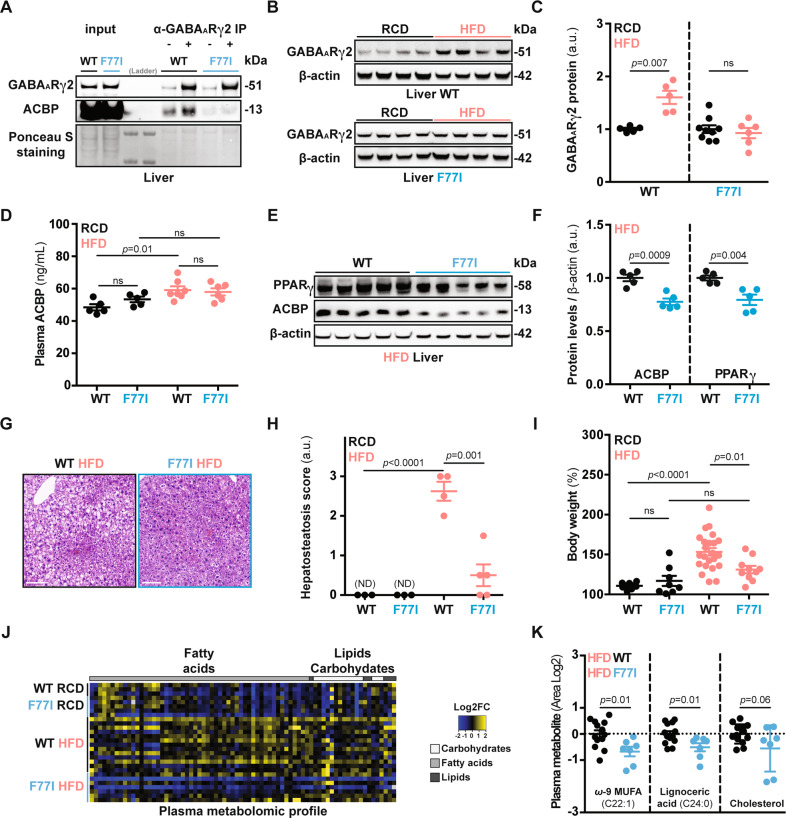
Metabolic effects of GABA_A_R – ACBP compromised signaling. **A** Co-immunoprecipitation (co-IP) describing the physical interaction between the ACBP and the GABA_A_R γ2 subunit in liver extracts from mice subjected to a high-fat diet (*n* = 3 per condition). **B** Liver representative immunoblot images of GABA_A_R γ2 subunit and β-actin proteins from mice receiving regular-chow (RCD) or high-fat diet (HFD) (1 month), densitometric quantification (*n* = 5 to 9 mice per condition) (**C**). **D** Plasma ACBP concentration measurement from WT and F77I mice fed with RCD or HFD (1 month) (*n* = 5 to 7 mice per condition). **E** Liver representative immunoblot images of PPARγ and ACBP proteins from WT and F77I mice receiving HFD (1 month), densitometric quantification (*n* = 5 mice per condition) (**F**). **G** Representative HE images of liver sections, **H** hepatosteatosis score quantification from WT or F77I mice after 1 month of HFD (bar: 50 μm, ND non-detected) (*n* = 3 to 5 mice per condition). **I** Body weight measurement from WT and F77I mice fed with RCD or HFD (*n* = 8 to 23 mice per group). **J** Heatmap representation of fatty acid, lipid, and carbohydrate plasma concentrations depicted as Area log2-fold change (Area Log2FC) from WT or F77I mice fed with RCD or HFD (1 month) (*n* = 3 to 13 mice per condition) followed by quantification of representative lipid metabolism-related metabolites (**K**). Results are displayed as whisker plots (with each dot representing one single mouse) including the mean ± SEM. For statistical analysis *p* values were calculated by two-tailed unpaired Student’s *t*-test (**F**, **K**) applying Welch correction (**C**), two-way ANOVA (**D**, **I**), or Mann–Whitney test (**H**). kDa kilodaltons, a.u. arbitrary units, ns non-significant, ND non-detected. See also Fig. S6.

Mutation of the γ2 subunit of the pentameric GABA_A_R (the phenylalanine in position 77 substituted by isoleucine, hereafter referred to as F77I) reportedly abolishes ACBP signaling [24, 42]. Accordingly, ACBP protein could be immunoprecipitated with the GABA_A_R γ2 subunit from wild-type mice (WT) but not with the γ2 subunit from mice that bear the *Gabrg2 F77I* allele in homozygosity (genotype: *Gabrg2*^*F77I/F77I*^) (Fig. 6A). *Gabrg2*^*F77I/F77I*^ mice are refractory to the hyperphagia induced by recombinant ACBP protein administration [10]. Therefore, we investigated whether GABA_A_R is coupled to lipoanabolism. HFD caused the upregulation of GABA_A_R γ2 WT protein but not of GABA_A_R γ2 F77I protein in the liver (Fig. 6B, C). Similarly, *Ob/Ob* mice (that are leptin-deficient and overeat a normal diet) [43] upregulated liver GABA_A_R γ2 protein together with an increase of ACBP and FASN, compared to their lean *Ob/T* counterparts (Fig. S6A-C). Even though HFD increased the plasma ACBP levels (irrespectively of the *Gabrg2* genetic background, Fig. 6D), the ACBP and PPARγ protein levels were downregulated in the livers of *Gabrg2*^*F77I/F77I*^ mice both over a short (1 month, Fig. 6E, F) and a long course (4 months, Fig. S6D, E) of HFD. In line with these observations, the HFD-induced triglyceride and cholesterol synthesis was decreased in the livers of *Gabrg2*^*F77I/F77I I*^ mice, suggesting compromised lipoanabolism (Fig. S6F–I). Of note, the aforementioned metabolic effects of *Gabrg2*^*F77I/F77I*^ mice were not related to hypophagia (Fig. S6J).

Given that ACBP signaling promotes a number of obesity-related features (including hepatosteatosis, weight gain, and hyperlipidemia) [9], we asked whether the neutralization of GABA_A_R—ACBP signaling would be sufficient to ameliorate obesity. Interestingly, when compared to their WT counterparts, HFD-fed *Gabrg2*^*F77I/F77I*^ mice exhibited reduced hepatosteatosis (Fig. 6G, H), were refractory to HFD-induced weight gain (Fig. 6I), and exhibited reduced circulating free fatty acids and cholesterol levels (Fig. 6J, K). Liver RNA-seq analyses revealed that several genes (including the obesity-promoting *Lcn2* gene) that were upregulated by HFD in GABA_A_R WT mice, failed to be induced in *Gabrg2*^*F77I/F77I*^ mice (Fig. S6K, L).

Altogether, these results confirm that GABA_A_R γ2 is an essential component of the feedforward circuitry that involves ACBP and PPARγ.

## Discussion

Our data support the existence of a tripartite obesogenic amplification loop that is activated in the liver of mice on HFD. This involves (i) the lipogenic and appetite-stimulatory factor ACBP, (ii) the ACBP receptor GABA_A_R, and [3] the TF PPARγ that transactivates the ACBP gene downstream of the ACBP-GABA_A_R interaction. The arguments pleading in favor of this trio can be summarized as follows:

First, ACBP is induced by HFD in various tissues, resulting in the increase of its plasma levels. Adipocyte-specific or whole-body knockout of ACBP, as well as its antibody-mediated neutralization, reduce HFD-induced lipoanabolism, hepatosteatosis, and hyperglycemia [9]. Moreover, neutralization of ACBP reduces the activation of PPARγ by HFD. Conversely, systemic injection of ACBP protein upregulates PPARγ in hepatocytes as it stimulates lipogenesis [9].

Second, HFD or overconsumption of a normal diet (in leptin-deficient *Ob/Ob* mice) increases the hepatic expression of the γ2 subunit of GABA_A_R. However, HFD fails to increase the expression of the γ2 subunit of a mutant GABA_A_R (F77I) that cannot bind ACBP. *Gabrg2*^*F77I/F77I*^ mice also fail to mount an orexigenic response to recombinant ACBP administration [10] and exhibit attenuated HFD-induced hepatosteatosis, as well as reduced PPARγ activation and ACBP expression, as compared to their WT counterparts.

Third, HFD increases the PPARγ binding to the *Acbp* gene promoter in murine livers. In vivo knockdown of *Pparg* reduces HFD-induced lipoanabolism, hepatosteatosis, and hyperglycemia, as it prevents the HFD-induced increase of ACBP. Conversely, direct stimulation of PPARγ with rosiglitazone, a thiazolidinedione antidiabetic that is well known for inducing weight gain in patients [44–46], resulted in upregulation of ACBP, and an ACBP-dependent increase in body mass.

The aforementioned results, obtained in vivo, are supported by in vitro experiments with human and mouse liver cell lines showing that PPARγ is required for the baseline expression of ACBP and that rosiglitazone and other PPARγ agonists induce ACBP expression by on-target effects. Moreover, strong correlative evidence obtained on human tissues supports the hypothesis that PPARγ is activated in obesity and is closely associated with ACBP expression in metabolically relevant tissues.

At a more speculative level, it appears intriguing that diet-induced weight gain is tied to the activation of phylogenetically ancient circuitries. In evolutionary terms, ACBP is the oldest appetite stimulator [8]. Indeed, ACBP is the sole protein to be secreted by unicellular fungi. In response to starvation, it stimulates sporulation, which is the only form of locomotion possible for such species, allowing them to find new nutrient resources [16, 47, 48]. Similarly, PPARγ appears to be a nutrient-responsive, ancient TF, orthologues of which have been identified in nematodes [49], fruit flies [50, 51], as well as in a variety of non-mammalian vertebrates including marine teleosts [52] and reptiles [53]. Of note, human-specific single nucleotide polymorphisms in *PPARG2* have been established during primate evolution [54], perhaps marking a human-specific proclivity to develop obesity [55]. Thus, in a theoretical scenario, body weight regulation by ACBP and PPARγ may have become connected at some step of animal phylogeny.

Of note, there is no orthologue of PPARγ in yeast, where ACBP acts on a G-protein coupled receptor (GPCR), STE3, which is also a pheromone receptor [16]. In mice, ACBP and its peptide fragment octadecaneuropeptide (ODN) can act on a pertussis toxin-inhibitable ODN-GPCR, as well as on GABA_A_R, which is not a GPCR but rather a ligand-activated chloride channel [56]. ACBP injected into the brain (intrathecally or into the hypothalamus) has anxiogenic and anorexigenic effects that can be blocked by ODN-GPCR inhibition [57, 58], contrasting with the GABA_A_R-mediated orexigenic effects of peripherally (intravenously or intraperitoneally) administered ACBP [10]. Since whole-body inactivation of ACBP and mutation of GABA_A_R have similar metabolic effects as peripherally injected anti-ACBP antibodies, it appears that the peripheral ACBP effects dominate over its central-nervous impact.

In this context, it should be noted that GABA, the natural ligand of GABA_A_R, has been implicated in obesogenic pathways outside of the nervous system. Thus, hepatic synthesis of GABA, catalyzed by GABA-transaminase, is upregulated in obese mice and persons, and its inhibition improves both hyperphagy and diabetes in mice [59]. GABA is also increased in the interscapular BAT of obese mice, and its oral supplementation with the drinking water suffices to worsen glucose intolerance in the context of HFD [60]. Since GABA cannot cross the blood–brain barrier [61], such effects should be mediated by peripheral (i.e., non-cerebral) GABA receptors. Nonetheless, additional experimentation involving tissue- and cell-type-specific ablation of GABA_A_R subunits is necessary to formally determine whether central or peripheral GABA_A_R signaling dictates the role of GABA and ACBP in metabolic regulation.

Irrespective of the aforementioned uncertainties, it appears that the tripartite feedforward loop driving obesity that we delineate here offers several pharmacological targets for therapeutic intervention. First, active or passive immunization against ACBP may be envisaged to induce neutralizing anti-ACBP autoantibodies or to inject subcutaneous depots of recombinant anti-ACBP antibodies, respectively. This latter technology has been successfully employed for administering an anti-RANKL antibody (denosumab) to women at risk of osteoporosis [62], indicating its utility for neutralizing harmful cytokines in a clinical setting. Second, efforts might be launched to identify small molecules (that ideally would not cross the blood–brain barrier, yet would be orally available) to block the interaction of ACBP with the γ2 subunit of GABA_A_R. The advantage of such an approach is that it involves the competitive disruption of a classical (“druggable”) receptor-ligand interaction. Finally, on a third level, the development of PPARγ antagonists may be attempted. In favor of this latter possibility, it appears that a loss-of-function polymorphism in human PPARγ2 (P12A) correlates with reduced body mass index and blood glucose levels [63]. Pharmacological PPARγ antagonists have already been shown to have anti-obesity and antidiabetic activity in preclinical models [64]. However, the identification of small molecules that specifically block the activity of TFs is notoriously difficult [65].

In sum, our present work identifies three molecules, (i) the obesogenic factor ACBP, (ii) the ACBP receptor GABA_A_R, and (iii) the ACBP-transactivating TF PPARγ, as elements of a vicious amplification loop that likely contributes to the pathogenesis of obesity and its comorbidities. Future investigation will delineate how this feedforward loop crosstalks with other obesogenic mechanisms including neuropsychiatric, endocrine, metabolic, inflammatory, immune, and microbial circuitries that determine the close-to-irreversible nature of excessive weight gain.

## Supplementary information

Reproducibility checklist

ChIPseq PPARg raw data Figure 1D

Metabolomics raw data

Supplemental Figures legends

Figure S1

Figure S2

Figure S3

Figure S4

Figure S5

Figure S6

## Data Availability

The RNA-seq data reported in this study are available at the Gene Expression Omnibus (GEO) under accessions: GSE191004.
