# Correction: An obesogenic feedforward loop involving PPARγ, acyl-CoA binding protein and GABA receptor

**DOI:** 10.1038/s41419-022-04884-9

**Published:** 2022-05-03

**Authors:** Gerasimos Anagnostopoulos, Omar Motiño, Sijing Li, Vincent Carbonnier, Hui Chen, Valentina Sica, Sylvère Durand, Mélanie Bourgin, Fanny Aprahamian, Nitharsshini Nirmalathasan, Romain Donne, Chantal Desdouets, Marcelo Simon Sola, Konstantina Kotta, Léa Montégut, Flavia Lambertucci, Didier Surdez, Grossetête Sandrine, Olivier Delattre, Maria Chiara Maiuri, José Manuel Bravo-San Pedro, Isabelle Martins, Guido Kroemer

**Affiliations:** 1 Centre de Recherche des Cordeliers, Equipe labellisée par la Ligue contre le cancer, Université de Paris, Sorbonne Université, Inserm U1138, Institut Universitaire de France, Paris, France; 2 Metabolomics and Cell Biology Platforms, Institut Gustave Roussy, Villejuif, France; 3 Faculté de Médecine, Université de Paris Saclay, Kremlin Bicêtre, France; 4 Department of Experimental and Health Sciences, Pompeu Fabra University (UPF), Barcelona, Spain; Center for Networked Biomedical Research in Neurodegenerative Diseases (CIBERNED), Madrid, Spain; 5 Laboratory of Proliferation, Stress and Liver Physiopathology, Centre de Recherche des Cordeliers, 75006 Paris, France; 6 Centre de Recherche des Cordeliers, INSERM, Sorbonne Université, Université de Paris, F-75006 Paris, France; 7 INSERM U1163, Institut Imagine, Paris, France; 8 INSERM U830, Équipe Labellisée LNCC, Diversity and Plasticity of Childhood Tumors Lab, PSL Research University, SIREDO Oncology Centre, Institut Curie Research Centre, 75005 Paris, France; 9 Bone Sarcoma Research Laboratory, Balgrist University Hospital, University of Zurich, Zurich, Switzerland; 10 INSERM U830, Équipe Labellisée LNCC, Diversity and Plasticity of Childhood Tumors Lab, PSL Research University, SIREDO Oncology Centre, Institut Curie Research Centre, 75005 Paris, France; 11 Unité de Génétique Somatique, Service d’oncogénétique, Institut Curie, Centre Hospitalier, 75005 Paris, France; 12 Facultad de Medicina, Departamento de Fisiología, Universidad Complutense de Madrid, Madrid, Spain; 13 Pôle de Biologie, Hôpital Européen Georges Pompidou, AP-HP, Paris, France

**Keywords:** Cell biology, Diseases

Correction to: *Cell Death and Disease* 10.1038/s41419-022-04834-5, published online 18 April 2022

The original version of this article unfortunately contained an error in an author name. instead of “Bravo-SanPedro”, the name should be “Bravo-San Pedro” with a space between “San” and “Pedro”. We apologize for this error. The original article has been corrected.

