# Supplementary material for: An obesogenic feedforward loop involving PPARγ, acyl-CoA binding protein and GABAA receptor
Source: Cell Death Dis. 2022 Apr 18;13(4):356. doi: 10.1038/s41419-022-04834-5 (PMC9016078; doi:10.1038/s41419-022-04834-5)
Supplement: Supplementary file 4 — Supplemental Figures legends [file 41419_2022_4834_MOESM4_ESM.docx]

**Supplemental Figure Legends**

**Figure S1. Related to Figure 1.**

Heatmap representation of correlation (R) between *ACBP* mRNA expression and the mRNA expression of several factors in various organs of human **(A)**, mouse **(B)**, and rat **(C)** origin.

**(D)** Binding motif analysis of human *ACBP* gene including characterized and putative TFs implicated in *ACBP* gene expression (Genome browser, ENCODE database).

**(E)** *ACBP* and *PPARG* mRNA expression measurements in HepG2 cells under negative control (Ctr), *ACBP*-, and *PPARG*-silencing conditions (n=4). Results are displayed as whisker plots (with each dot representing one biological replicate) including the mean ± SEM.

Results are displayed as heatmap representations **(A-C)** and whisker plots (with each dot representing one single mouse) including the mean ± SEM **(E)**. For statistical analysis, *p*-values were calculated by Bravais-Pearson **(A,B)** and Kendal **(C)** correlation, or one-way ANOVA **(E)**. a.u.: arbitrary units.

**Figure S2. Related to Figure 2.**

**(A)** Representative immunoblot images of ACBP and β-actin proteins in Hepa1-6 and Hep55.1c cells treated with Vehicle or Rosiglitazone (Rosi) for 48 h, densitometric quantification (n=4) **(B,C)**.

**(D)** *Acbp* mRNA expression measurement in epididymal white adipose tissue (eWAT) extracts obtained from mice receiving Vehicle or Rosi (5 days) (n=10 to 13 mice per condition).

**(E)** Representative immunoblot images of PPARγ and ACBP and β-actin proteins in eWAT obtained from mice receiving Vehicle or Rosi (5 days), densitometric quantification (n=5 to 8 mice per condition) **(F)**.

Results are displayed as whisker plots (with each dot representing one *in vitro* biological replicate or one single mouse) including the mean ± SEM. For statistical analyses, *p*-values (indicating statistical comparisons with the control condition) were calculated by two-tailed unpaired Student’s t test **(B,C,D,F)**. a.u.: arbitrary units.

**Figure S3. Related to Figure 3.**

**(A)** Schematic representation of the experimental setup. Adult male mice received intraperitoneally (i.p.) the following drugs (5 days): negative control (Vehicle), RXRα agonist Bexarotene (Bex), or RXRα antagonist HX531.

**(B)** Plasma ACBP concentration (n=17 to 20 mice per condition), and **(C)** body weight (n=15 mice per condition) measurements in mice receiving regular-chow (RCD) or high-fat diet (HFD) for 1 month.

**(D)** Schematic representation of whole-body inducible *Pparg* knockout mice (*ubi:Pparg KO*) and their wild-type littermates (*ubi:Pparg WT*).

**(E)** Liver representative immunoblot images of FASN, PPARγ ACBP and GAPDH proteins from *ubi:Pparg WT* and *ubi:Pparg KO* mice receiving RCD (4 months), densitometric quantification (n=5 to 6 mice per condition).

Results are displayed as whisker plots (with each dot representing one single mouse) including the mean ± SEM. For statistical analyses, *p*-values (indicating statistical comparisons with the control condition) were calculated by two-tailed unpaired Student’s t test **(F)** applying Welch correction **(B,C)**. a.u.: arbitrary units,

**Figure S4. Related to Figure 4.**

**(A)** Schematic representation of the experimental setup. Wild-type mice received retro-orbitally adeno-associated viral vectors (AAV8) delivering negative control (*AAV8 [CRISPR_Rosa26_ ; Cas9*]) or *Pparg*-specific (*AAV8 [CRISPR_Pparg_ ; Cas9*]) CRISPR/Cas9 components in order generate liver-specific PPARγ knock down (KD) genetic background. The control mice (PPARγ WT) and their PPARγ KD counterparts received RCD or HFD for 2 months.

**(B)** Heatmap representation of plasma metabolite concentrations depicted as Area log2 from PPARγ WT and PPARγ KD mice fed with RCD or HFD (n=8 to 10 mice per condition). Quantification graphs of representative metabolites [carnitine group **(C)**, unsaturated fatty acid group **(D)**, and saturated fatty acid group **(E)**] obtained from plasma of WT or PPARγ KD mice receiving RCD or HFD (n=8 to 10 mice per condition).

Results are displayed as heatmap representation **(B)** and whisker plots (with each dot representing one single mouse) including the mean ± SEM **(C-E)**. For statistical analyses, *p*-values were calculated by Klustal-Wallis test **(C-E)**.

**Figure S5. Related to Figure 5.**

**(A)** Representative liver hematoxylin-eosin (HE) histological sections from mice receiving isotype control (Iso) or α-ACBP (under regular or high-fat diet nutritional regimen) followed by NAFLD (n=5 to 10 mice per condition) **(B)**, and inflammation score quantification (n=4 to 10 mice per condition) **(C)**.

**(D)** β-hydroxybutyrate (n=10 mice per condition) and **(E)** leptin (n=4 to 12 mice per condition) concentration measurements from blood obtained from mice receiving Iso or α-ACBP.

**(F)** Schematic representation of adipocyte-specific *Acbp* knockout mice (*adipo:Cre +/+ ; Acbp KO*) and their wild-type littermates (*adipo:Cre -/- ; Acbp WT*).

**(G)** eWAT representative immunoblot images of ACBP and β-actin proteins from adipocyte-specific ACBP wild-type and knockout mice receiving RCD (6 weeks), densitometric quantification (n=6 to 10 mice per condition) **(H)**.

Results are displayed as whisker plots (with each dot representing one single mouse) including the mean ± SEM. For statistical analyses, *p*-values (indicating statistical comparisons with the control condition) were calculated by Mann-Whitney test **(B)**, two-way ANOVA **(C-E)** or two-tailed unpaired Student’s t test applying Welch correction **(H)**. Bar: 50 μm **(A)**. a.u.: arbitrary units.

**Figure S6. Related to Figure 6.**

**(A)** Body weight measurement of wild-type *Ob/T* mice (lean) compared to their leptin-deficient *Ob/Ob* (obese) hyperphagic genetic counterparts (n=10 mice per condition).

**(B)** Representative immunoblot images of FASN, GABA_A_R γ2, ACBP and β-actin proteins in liver extracts obtained from *Ob/T* or *Ob/Ob* mice, densitometric quantification (n=7 to 8 mice per condition) **(C)**.

**(D)** Representative immunoblot images of PPARγ and GAPDH proteins in liver extracts obtained from WT or F77I mice receiving HFD for a prolonged period (HFD_prolonged_: 18 weeks), densitometric quantification (n=5 to 9 mice per group) **(E)**.

Measurement of **(F)** glycerol 3-phosphate and **(G)** cholesterol levels in liver extracts obtained from WT or F77I mice RCD or HFD (18 weeks) (n=3 to 8 mice per condition).

**(H)** Representative immunoblot images of PPARγ, ACBP and β-actin proteins in epididymal white adipose tissue extracts obtained from WT or F77I mice fed with HFD, densitometric quantification (n=6 to 7 mice per condition) **(I)**.

**(J)** Cumulative food intake measurement of WT or F77I mice receiving RCD or HFD (n=5 to 12 mice per condition).

**(K)** Heatmap representation of liver mRNA transcripts [as log2-fold change (Log2FC)] which were significantly upregulated in WT HFD group (but not altered in the F77I) (n = 3 to 10 mice per condition). Gene ontology analysis of the differentially expressed genes (HFD WT vs HFD F77I, *p*<0.05): Biological Process (BP); gene groups implicated in inflammation are indicated in red **(L)**.

Results are displayed as heatmap representation **(K)** and whisker plots **(A,C,E-G,I,J)** (with each dot representing one single mouse) including the mean ± SEM. For statistical analysis *p*-values were calculated by two-tailed unpaired Student’s t-test **(C,I)** applying Welch correction **(A,E)**, or two-way ANOVA **(F,G,J)**. g: gram, a.u.: arbitrary units, ns: non-significant, kDa: kilodaltons, BP: biological process.
