# Supplementary figures and images for: An obesogenic feedforward loop involving PPARγ, acyl-CoA binding protein and GABAA receptor
Source: Cell Death Dis. 2022 Apr 18;13(4):356. doi: 10.1038/s41419-022-04834-5 (PMC9016078; doi:10.1038/s41419-022-04834-5)

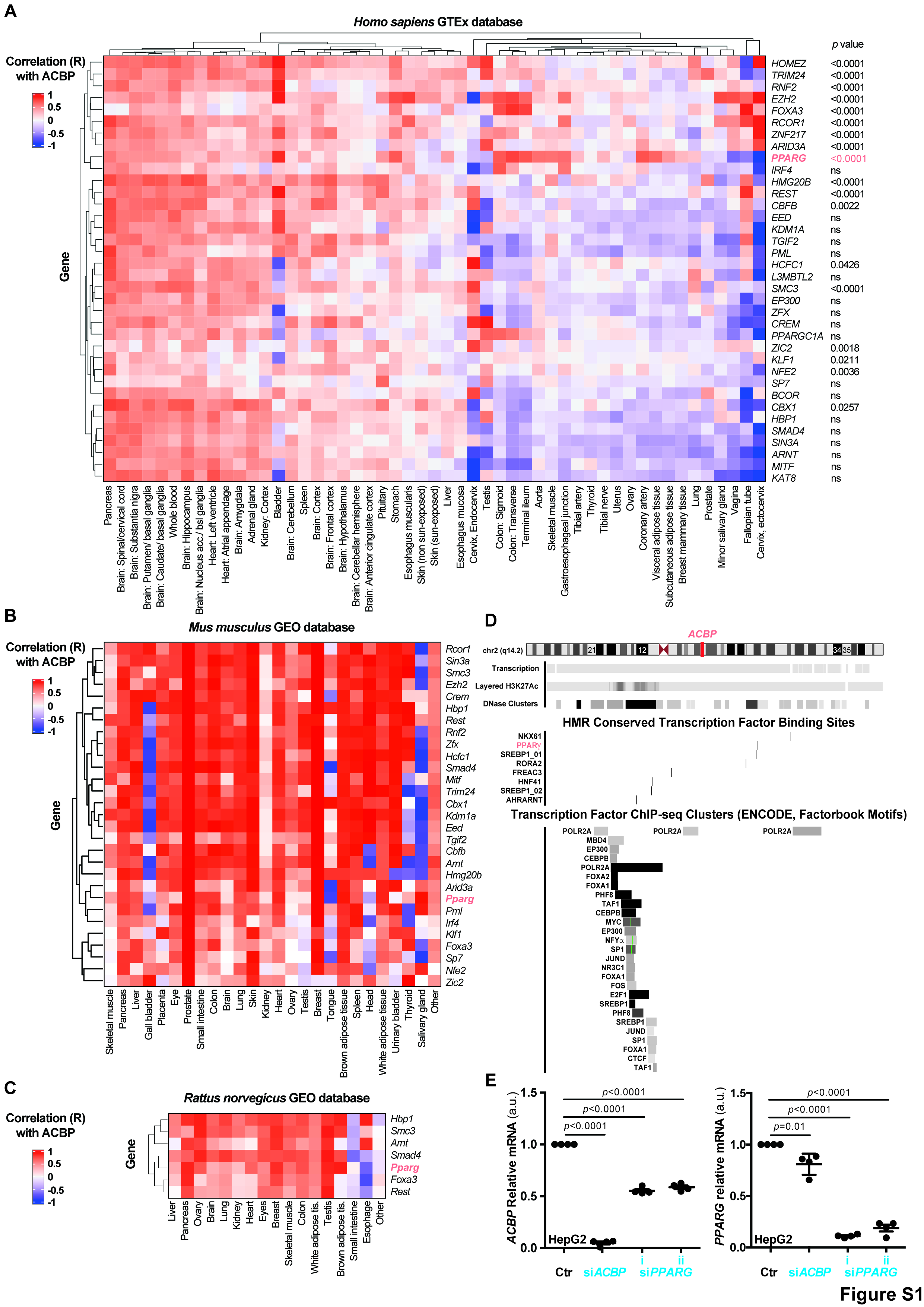

Supplement: Supplementary file 5 — Figure S1 [file 41419_2022_4834_MOESM5_ESM.tif]

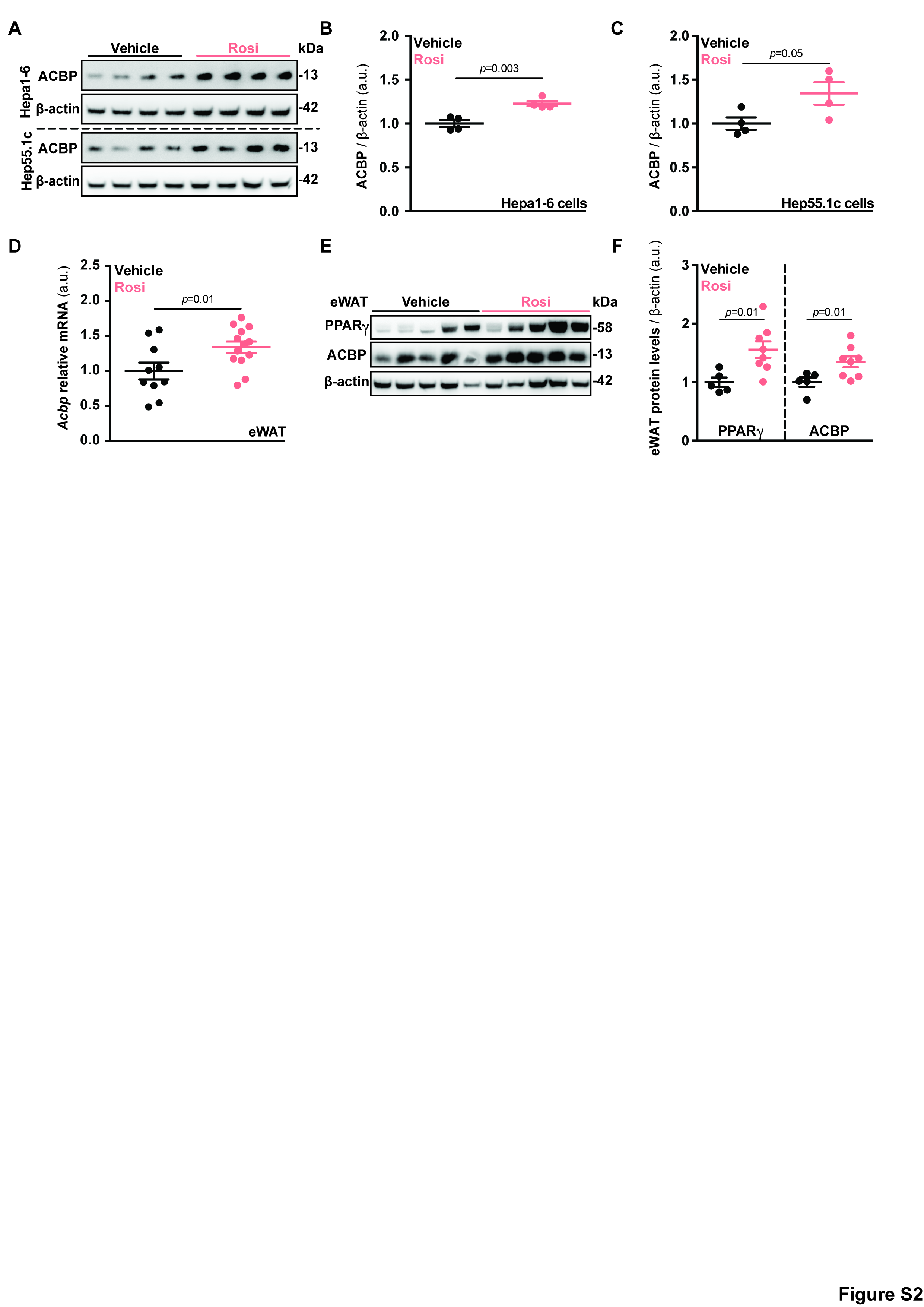

Supplement: Supplementary file 6 — Figure S2 [file 41419_2022_4834_MOESM6_ESM.tif]

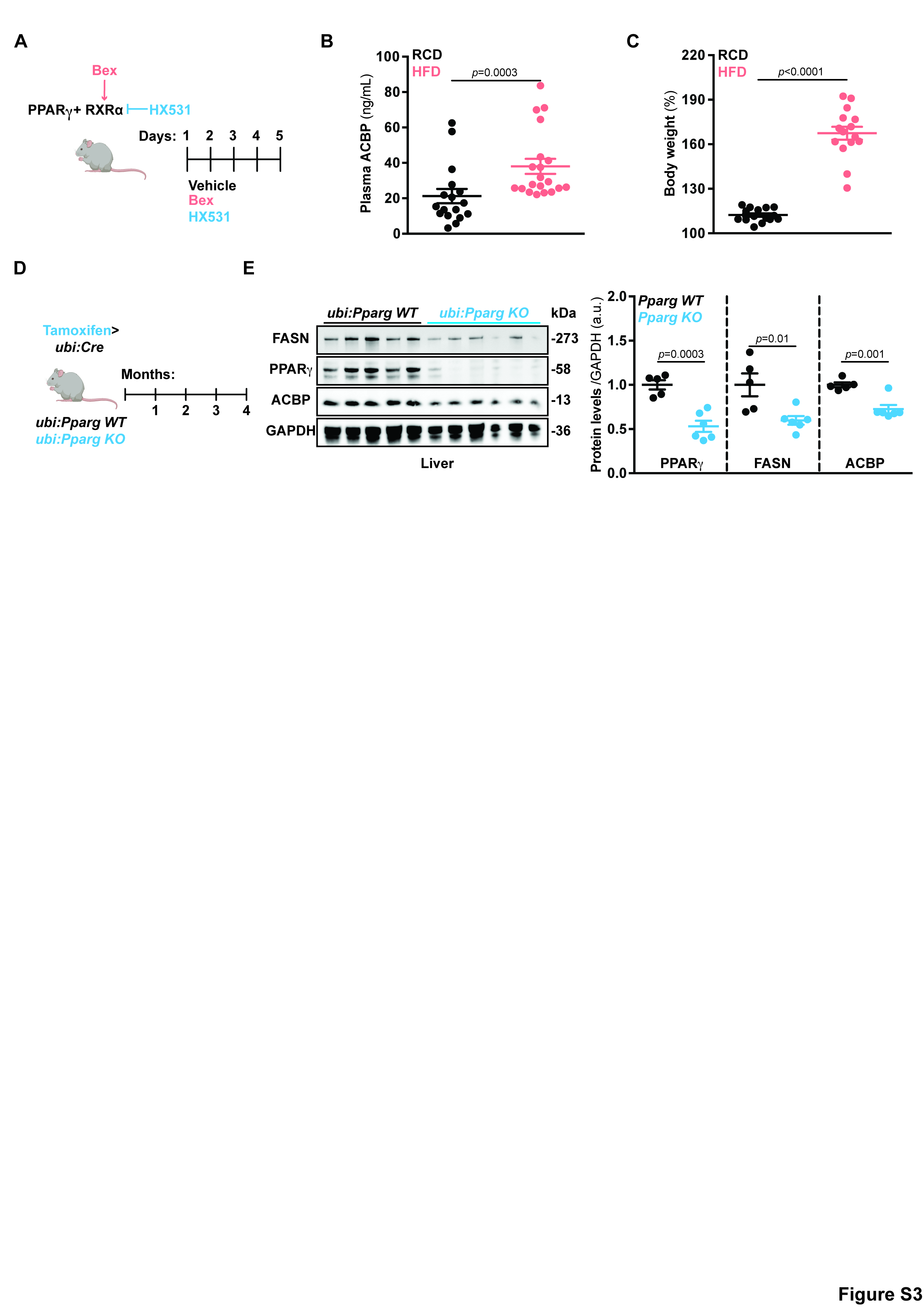

Supplement: Supplementary file 7 — Figure S3 [file 41419_2022_4834_MOESM7_ESM.tif]

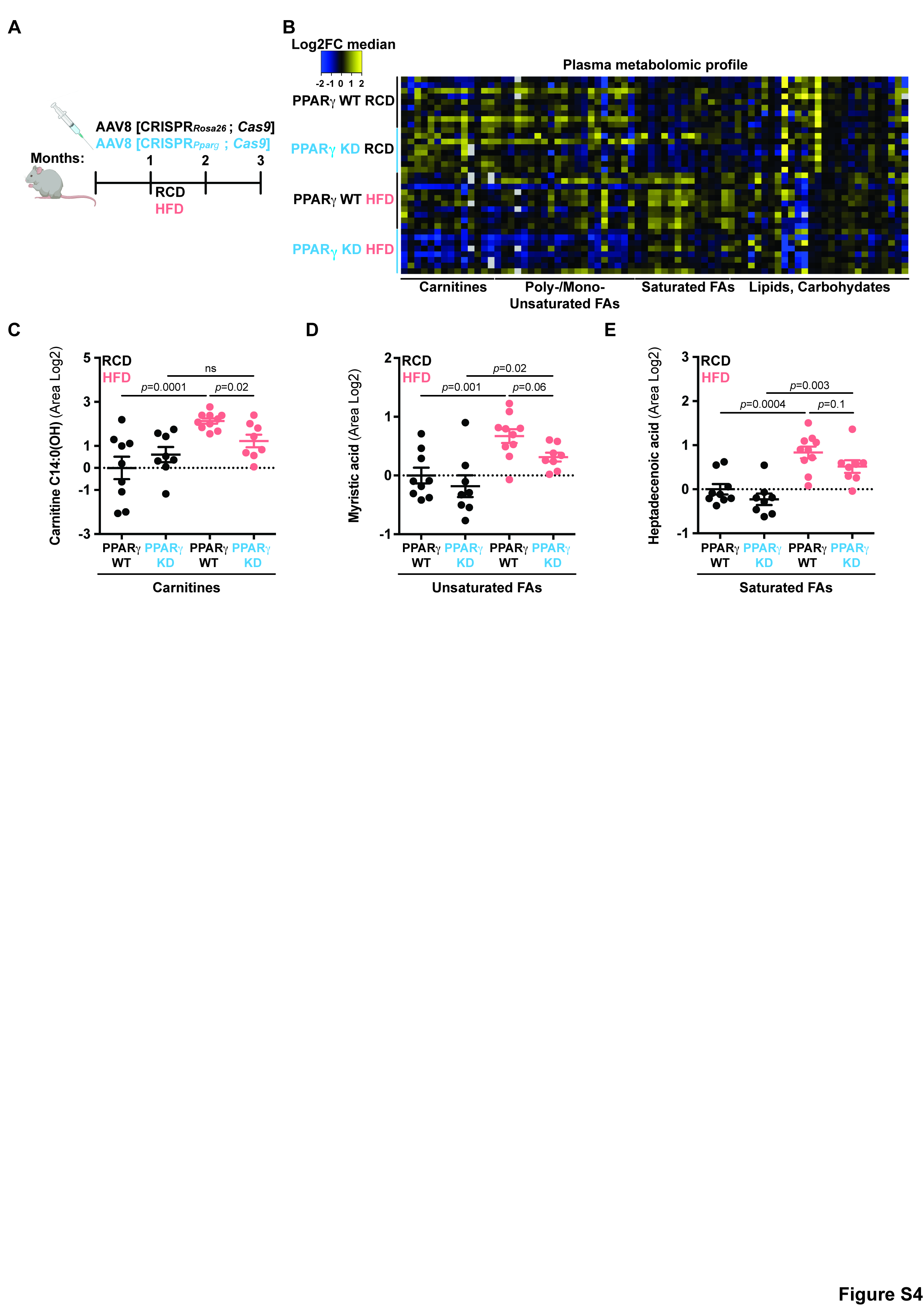

Supplement: Supplementary file 8 — Figure S4 [file 41419_2022_4834_MOESM8_ESM.tif]

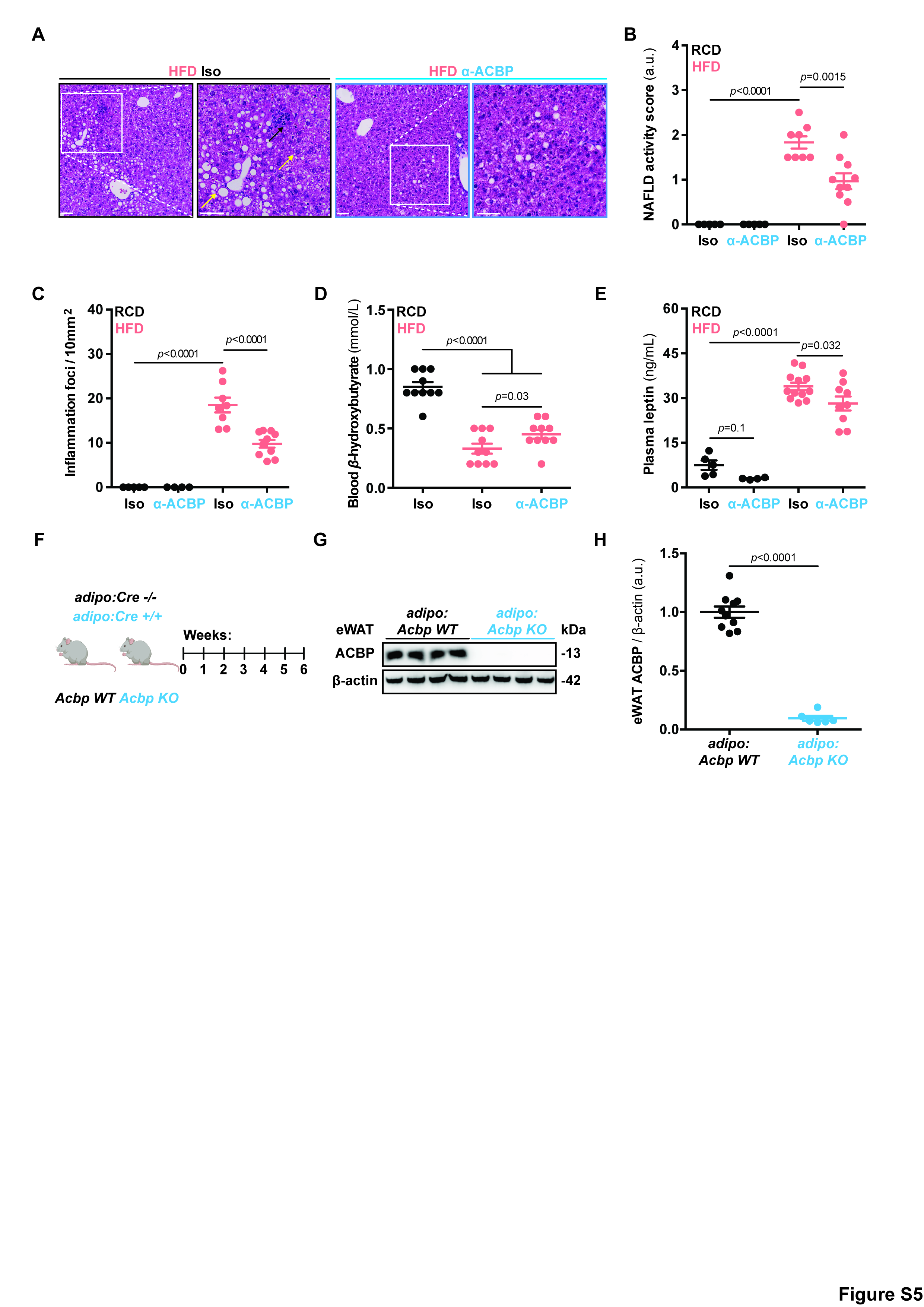

Supplement: Supplementary file 9 — Figure S5 [file 41419_2022_4834_MOESM9_ESM.tif]

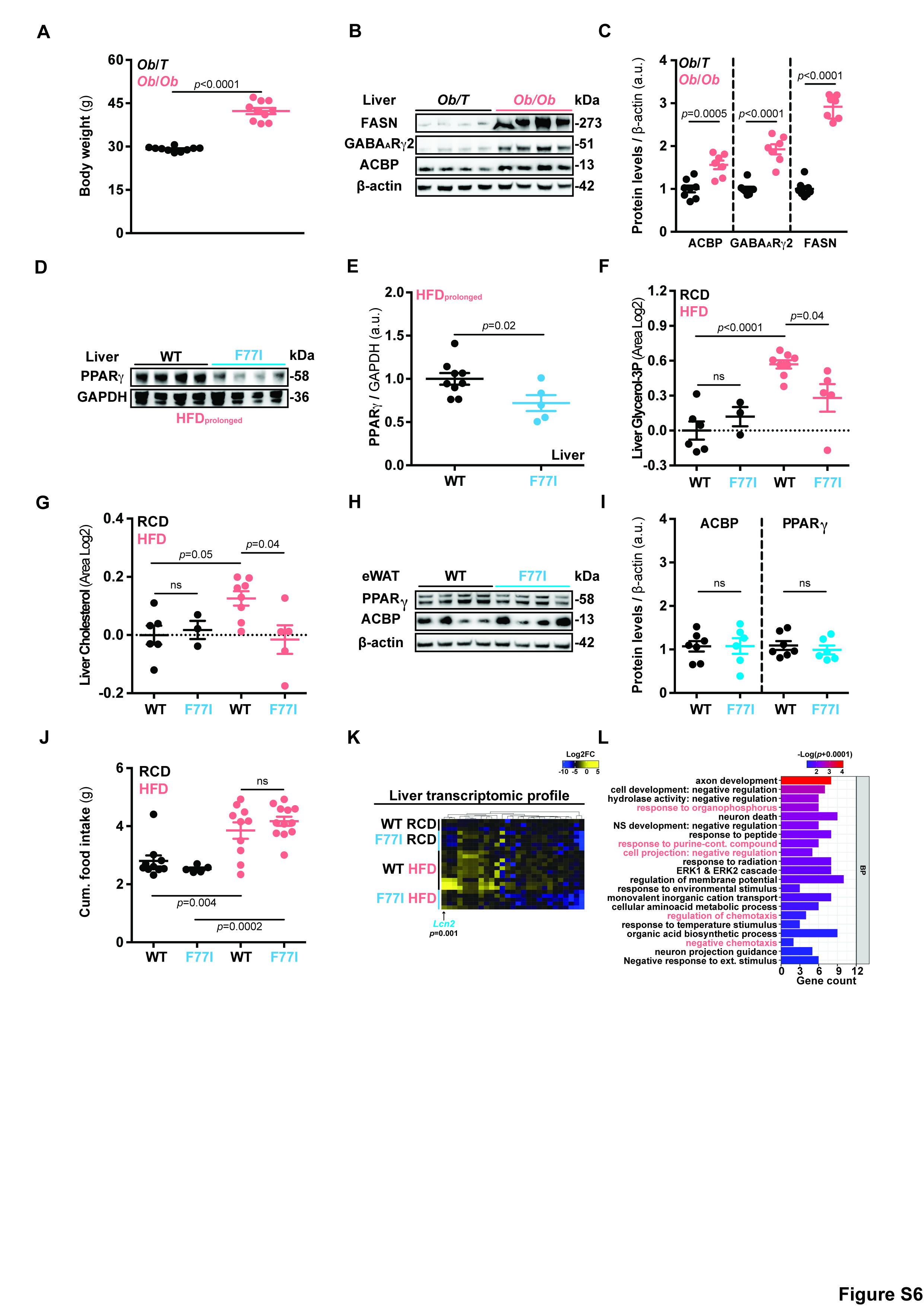

Supplement: Supplementary file 10 — Figure S6 [file 41419_2022_4834_MOESM10_ESM.tif]
